# Characterizing the Prevalence of Obesity Misinformation, Factual Content, Stigma, and Positivity on the Social Media Platform Reddit Between 2011 and 2019: Infodemiology Study

**DOI:** 10.2196/36729

**Published:** 2022-12-30

**Authors:** Catherine C Pollack, Jennifer A Emond, A James O'Malley, Anna Byrd, Peter Green, Katherine E Miller, Soroush Vosoughi, Diane Gilbert-Diamond, Tracy Onega

**Affiliations:** 1 Department of Biomedical Data Science Geisel School of Medicine at Dartmouth Lebanon, NH United States; 2 Department of Epidemiology Geisel School of Medicine at Dartmouth Lebanon, NH United States; 3 Department of Pediatrics Geisel School of Medicine at Dartmouth Lebanon, NH United States; 4 The Dartmouth Institute for Health Policy and Clinical Practice Hanover, NH United States; 5 Department of Computer Science Dartmouth College Hanover, NH United States; 6 Department of Medicine Geisel School of Medicine at Dartmouth Lebanon, NH United States; 7 Department of Population Health Sciences Huntsman Cancer Institute University of Utah Salt Lake City, UT United States

**Keywords:** obesity, misinformation, social stigma, social media, Reddit, natural language processing

## Abstract

**Background:**

Reddit is a popular social media platform that has faced scrutiny for inflammatory language against those with obesity, yet there has been no comprehensive analysis of its obesity-related content.

**Objective:**

We aimed to quantify the presence of 4 types of obesity-related content on Reddit (misinformation, facts, stigma, and positivity) and identify psycholinguistic features that may be enriched within each one.

**Methods:**

All sentences (N=764,179) containing “obese” or “obesity” from top-level comments (n=689,447) made on non–age-restricted subreddits (ie, smaller communities within Reddit) between 2011 and 2019 that contained one of a series of keywords were evaluated. Four types of common natural language processing features were extracted: bigram term frequency–inverse document frequency, word embeddings derived from Bidirectional Encoder Representations from Transformers, sentiment from the Valence Aware Dictionary for Sentiment Reasoning, and psycholinguistic features from the Linguistic Inquiry and Word Count Program. These features were used to train an Extreme Gradient Boosting machine learning classifier to label each sentence as 1 of the 4 content categories or other. Two-part hurdle models for semicontinuous data (which use logistic regression to assess the odds of a 0 result and linear regression for continuous data) were used to evaluate whether select psycholinguistic features presented differently in misinformation (compared with facts) or stigma (compared with positivity).

**Results:**

After removing ambiguous sentences, 0.47% (3610/764,179) of the sentences were labeled as misinformation, 1.88% (14,366/764,179) were labeled as stigma, 1.94% (14,799/764,179) were labeled as positivity, and 8.93% (68,276/764,179) were labeled as facts. Each category had markers that distinguished it from other categories within the data as well as an external corpus. For example, misinformation had a higher average percent of negations (β=3.71, 95% CI 3.53-3.90; *P*<.001) but a lower average number of words >6 letters (β=−1.47, 95% CI −1.85 to −1.10; *P*<.001) relative to facts. Stigma had a higher proportion of swear words (β=1.83, 95% CI 1.62-2.04; *P*<.001) but a lower proportion of first-person singular pronouns (β=−5.30, 95% CI −5.44 to −5.16; *P*<.001) relative to positivity.

**Conclusions:**

There are distinct psycholinguistic properties between types of obesity-related content on Reddit that can be leveraged to rapidly identify deleterious content with minimal human intervention and provide insights into how the Reddit population perceives patients with obesity. Future work should assess whether these properties are shared across languages and other social media platforms.

## Introduction

Social media has become a ubiquitous component of everyday life. A recent study suggested that 72% of Americans use social media, including 84% of those aged between 18 and 29 years, 81% of those aged between 30 and 49 years, and 73% of those aged between 50 and 64 years [1]. Although social media promises to foster meaningful connections between individuals around the world, it has been exploited to spread misinformation and disinformation on a variety of topics, from the 2016 presidential election to the COVID-19 pandemic [2,3]. The widespread use of social media presents an ideal medium to study the discourse surrounding these geopolitical and health topics, as well as other topics of concern. However, much of this work has focused on international infectious disease outbreaks and vaccines, with less research dedicated to understanding misinformation regarding chronic diseases such as obesity [4].

Over 42% of adults in the United States have obesity, with rates steadily increasing since the early 2000s [5]. Individuals with obesity may experience weight-related stigma, a phenomenon whereby individuals are ascribed negative traits (such as laziness) “due” to their weight [6-8]. Exposure to stigma has been associated with adverse physical, mental, and emotional health outcomes, and this effect is amplified by social media; for example, studies have shown that social media use is correlated with concerns over body image [9,10]. Social media–based studies on stigma have predominately focused on Facebook, Twitter, and Instagram, leaving a gap in understanding how stigma (and other deleterious content, such as misinformation) manifests on other platforms [11-13]. Furthermore, the aforementioned platforms are not typically used anonymously, which may differentially affect behaviors. In contrast, Reddit is an anonymized content aggregation platform with over 52 million daily active users as of January 2021 [14]. The platform consists of over 100,000 different “subreddits,” which are smaller communities that are themed around a given topic. Subreddits range from more general communities for news and science to highly specific subreddits such as r/bodyweightfitness (a subreddit dedicated to sharing workout routines with 2.3 million subscribers as of October 2021) or r/eatcheapandhealthy (a subreddit dedicated to how to eat healthy foods on a budget with 3.6 million subscribers as of October 2021) [15]. Users can engage with these subreddits either by creating a post or by commenting on another user’s post. These comments can also be commented upon, creating a branching comment section.

Previous research has leveraged content on specific subreddits to model predictors of successful weight loss [16,17]. These communities (such as r/loseit, which has 3 million subscribers as of October 2021) are designed to provide a space for individuals to seek motivation, to ask questions about weight loss, or to share their experiences. Other parts of the platform are not as supportive, and Reddit has historically faced scrutiny for the abundance of stigma on the platform. Most notable were concerns over the former subreddit r/fatpeoplehate, which was dedicated to ridiculing individuals with obesity. This subreddit was banned in 2015 during a push by the company to remove hate speech from the platform. An evaluation of this ban found that it was effective in reducing the amount of hate speech on Reddit that was directed toward individuals with obesity, including those who were previously active members of the banned subreddit [18]. However, this study was limited in scope, and there are no studies that comprehensively evaluate the presence of weight-related stigma or misinformation on the platform. Similarly, there are no studies that evaluate the presence of body positivity or factual content across the entire platform.

**Figure 1:**
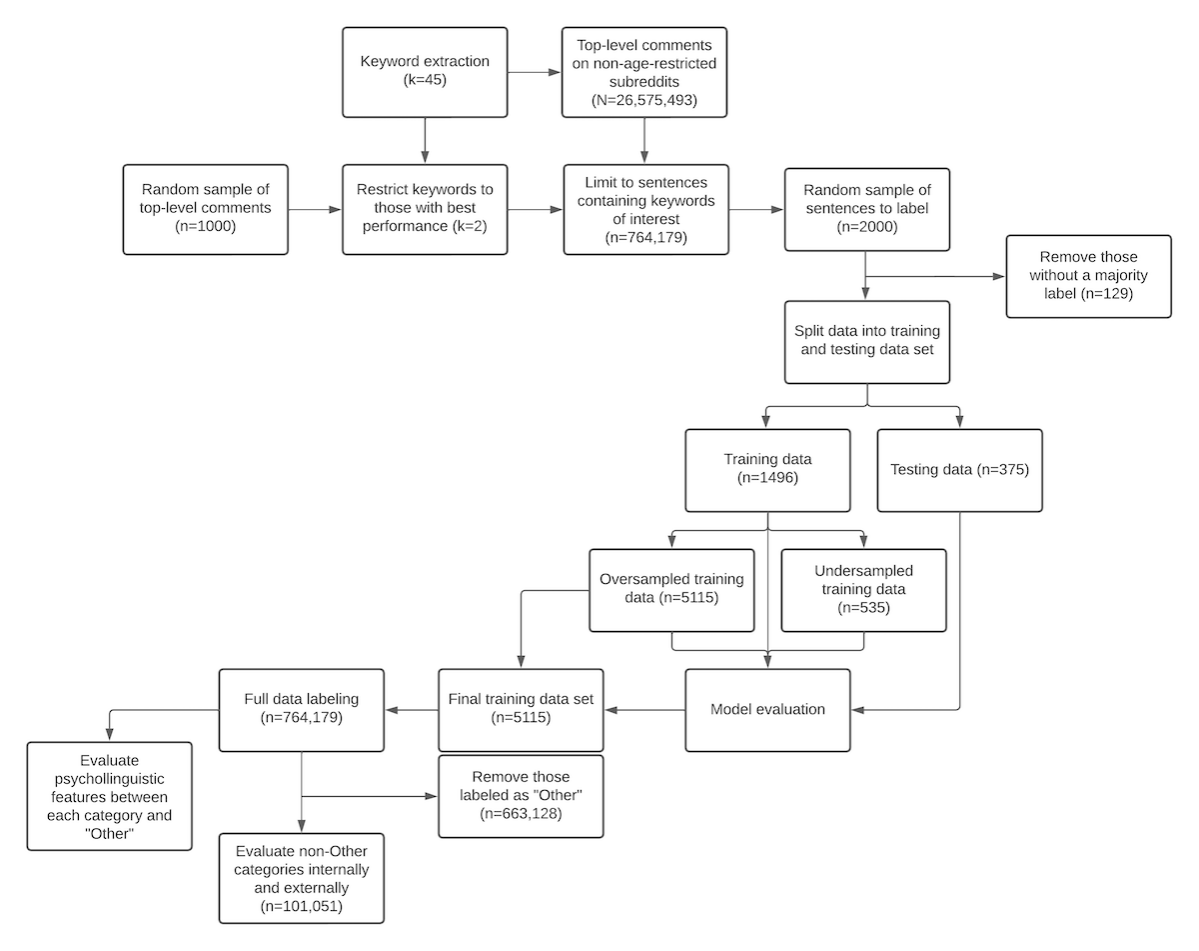
Flow diagram of process described in the Methods section. k: keyword.

The purpose of this study was to characterize obesity-related content on Reddit. To do this, a semiautonomous pipeline was created that leveraged a set of psycholinguistic and semantic features to differentiate the 4 categories of interest: misinformation, factual content, stigma, and positivity (Figure 1). Briefly, this pipeline involved extracting a series of sentences containing “obese” or “obesity” from a broader pool of comments; manually assigning a category to a small, random subset; extracting a candidate set of features; identifying the best model and feature set based on performance on the labeled subset; and using the identified model and feature set to automatically label the entire set of obesity-specific data. Following this, a statistical analysis was performed to evaluate whether there were distinct types of features that were either enriched or underrepresented within each category of interest. All categories were compared against an external benchmark and sentences labeled as other, and each “pair” of categories (ie, fact vs misinformation and positivity vs stigma) was internally compared.

## Methods

### Keyword Generation, Evaluation, and Data Collection

Keywords were derived from “obesity” and “diet” via colloquial terminology (eg, “chubby”), diet trends (eg, “keto”), and the National Institutes of Health National Library of Medicine Medical Subject Headings database (Multimedia Appendix 1) [19]. This process generated an initial list of 45 keywords that was used to curate all top-level Reddit comments (ie, comments that were made directly on a post and not another user’s comment) via Pushshift, a Reddit archive updated monthly [20]. Posts were excluded because they often contain video or image content that could not be evaluated in this study. Comments were limited to those made on non–age-restricted subreddits between 2011 and 2019, resulting in an initial corpus of 26,575,493 comments.

To evaluate keyword selection, a separate set of sentences from 1000 top-level Reddit comments were randomly collected and labeled by 1 member of the research team as to whether one of the keywords was included in the comment and, if so, whether the use of the word had a connotation related to obesity, nutrition, or weight loss. Of the 1000 sentences, 7 were “true positives” (ie, contained a keyword in a sentence that was related to obesity), 8 were “false positives” (ie, contained a keyword in a sentence unrelated to obesity), 7 were “false negatives” (ie, the sentence was related to obesity but did not include one of the keywords of interest), and the remaining were “true negatives” (ie, the sentence was not related to obesity and did not contain a keyword of interest). This resulted in high accuracy and specificity but low precision (ie, positive predictive value) and recall (ie, sensitivity; Multimedia Appendix 2). Consequentially, all analysis was restricted to only sentences containing the words “obese” or “obesity” (n=764,179 sentences across 689,447 comments after the removal of duplicates) to ensure that sentences included in the final analysis were related to the topic of interest. To assess the validity of this restriction, 100 random comments were selected for each keyword and evaluated as to whether they were related to the research question. Although 96% of comments containing obese and obesity were related to the research question, this was only true for 38% of comments for all keywords. Consequently, this restriction was considered sufficient.

### Data Labeling

Three research assistants (RAs) trained by researchers with expertise in obesity medicine independently labeled 2000 random sentences in the analytic data set as misinformation, fact, stigma, positivity, or other. Misinformation and factual content were distinguished in accordance with peer-reviewed guidelines identified by the American Board of Obesity Medicine and aligned with scientific literature [21]. Stigma was defined as derogatory language about individuals with obesity, including words such as “stupid,” “lazy,” or “dirty” [22]. Positivity was defined as affirmative language toward individuals with obesity or encouragement toward healthy weight loss (Multimedia Appendix 3).

In a form of hierarchical classification, the RAs were asked to assume a default label of fact or positivity unless the sentence contained misinformation (in which case they should label it as misinformation) or stigmatizing language (in which case they should label it as stigma). Sentences containing a mix of misinformation and stigmatizing language were labeled as stigma, while sentences containing both fact and positivity were labeled as fact. If the sentence was ambiguous or did not contain any type of information of interest, the RAs were instructed to label the sentence as other. A sentence was assigned a final label via an automated majority vote system if at least 2 RAs independently agreed on the label. If all 3 RAs disagreed on a label, the sentence was considered to not have a majority label. Using this method, 94% of sentences received a majority label. Fleiss κ was low (0.36), although this can occur even in instances where the agreement is high [23,24]. There were no significant psycholinguistic differences between posts that reached consensus and those that did not (Multimedia Appendix 3). Of the sentences receiving a majority label, 64% were labeled as other, 12% were labeled as fact, 9% were labeled as stigma, 5% were labeled as positivity, and 4% were labeled as misinformation.

### Feature Extraction

Four feature categories were extracted from sentences in the analytic data set. Basic word context was extracted using term frequency–inverse document frequency (TF-IDF), which weighs the number of times a word or phrase appears in a sentence by its commonality within all analyzed sentences [25]. For this implementation of TF-IDF, only bigrams (ie, 2-word phrases) were retained if they were in the training data and had a document frequency >1% and <75%. These thresholds were chosen to limit computational complexity by excluding highly rare terms (ie, those with a frequency <1%) and highly common terms (ie, those with a frequency >75%) that may not produce informative features. To extract further information on the text, a pretrained, case-sensitive Bidirectional Encoder Representations from Transformers (BERT) model was used to generate dense numerical vector representations of the input sentences (ie, sentence embeddings). BERT-based models expand on traditional natural language processing (NLP) models such as Word2Vec by preserving the context of the input sentence in addition to basic word choice [26]. Sentence sentiment was extracted using the Valence Aware Dictionary and Sentiment Reasoner (VADER). Designed specifically for social media data, VADER expands on traditional lexicon-based approaches by incorporating grammatical rules into its analysis, including the use of capitalization, punctuation, negation, and emojis. The output of VADER is the ratio of text that is characterized as positive, neutral, and negative. These values are then used to generate a normalized, weighted composite score that aims to capture the overall sentiment of the text within a single number. It is calculated by summing the adjusted valence of each word within the text and normalizing it such that it falls on a scale from −1 to 1. After this normalization, values ≤−0.05 were considered “negative,” values ≥0.05 were considered “positive,” and values between −0.05 and 0.05 were considered “neutral” [27,28]. Specific psycholinguistic features were evaluated through the Linguistic Inquiry and Word Count (LIWC) program, which identifies the percentage of the sentence that can be ascribed to 80 different categories, including functional words (eg, pronouns, adjectives, and numbers), social words (eg, female and male referents), and informal speech (eg, swear words and punctuation). The LIWC also includes 4 proprietary metrics related to analytic thinking (characterized as logical and hierarchical thought), clout (characterized as displaying social status or confidence), authenticity (characterized as displaying humbleness or vulnerability), and emotional tone (whereby lower values represent more negative emotion). Additional metrics can be found in other studies [29,30]. The psycholinguistic features included in the LIWC can provide valuable insights into the writer’s attitudes and perceptions toward the main topic of interest (such as obesity) [30]. Its utility in NLP tasks in the health domain on Reddit has been validated in other studies, including one that examined depression-related content on Reddit [31].

### Model Development and Evaluation

A series of machine learning classification models were repeatedly trained and tested on the subset of 2000 labeled sentences to determine which would perform best at labeling the full data set. Five initial models were selected based on their innate ability to perform multiclass classification, capture nonlinearity in data, and generally achieve successful out-of-the-box performance. These models were random forest, Extreme Gradient Boosting (XGBoost), support vector machine with a radial basis function kernel, multinomial naive Bayes, and multilayer perceptron. Two “dummy” classifiers were also used so that model performance metrics for the 5 candidate classifiers could be compared with classifiers that use basic rules to assign the final label. One dummy classifier (“stratified” model) predicted the final label based on the class distribution of the training set, while the other (“most frequent” model) assigned every sentence the most frequent label within the training set. If a more complex candidate model could not outperform both dummy classifiers, this would suggest that it could not identify an underlying pattern within the data that could inform label selection; in other words, it could not “learn” how to distinguish misinformation, facts, stigma, and positivity from the extracted linguistic and semantic features. All models were deployed using the default hyperparameters.

Each model was evaluated using 3 versions of an 80:20 train-test split of the original data that received a majority label. In the first version, the training data remained unchanged (train: n=1496; test: n=375). In the second version, all minority classes were oversampled to match the size of the majority class (train: n=5115). This oversampling involved random sampling with replacement from each minority class to create a balanced data set. In the third version, the majority class was downsampled to match the size of the smallest minority class (train: n=535). This process involved randomly selecting sentences from the majority class until a pool of sentences the same size as the smallest minority class was obtained. Both the second and third versions were performed to account for the unbalanced nature of the data; in all scenarios, the test data set remained unmodified so that an unbiased estimate of model performance could be calculated. All the features generated in *Feature Extraction* were first used in the models. The model and data set with the best performance were then subjected to forward variable selection to determine if a more parsimonious model could be generated without performance loss. The models were first trained using only 1 category of features (ie, TF-IDF, BERT, LIWC, or VADER). The feature with the best individual performance was then carried through to a secondary test that added additional features. This process was conducted iteratively until a model with all features was calculated or no future improvement was achieved. Model performance was also evaluated while varying the size of the training data to determine whether similar performance could be obtained with fewer labeled data. In all instances, model performance was evaluated using accuracy (ie, the proportion of sentences correctly assigned to their labeled class), average weight precision (ie, the weighted average across classes of the percentage of sentences assigned a given label that truly belong to that label), average weighted recall (ie, the weighted average across classes of the percentage of sentences of a given class assigned their correct label), and *F*_1_-score (ie, the harmonic mean of precision and recall). Figure 1 shows a flow diagram depicting this process.

### Statistical Analysis

Model feature importance was assessed by evaluating both the top 10 most frequent features across all trees used to split variables, as well as the top 10 features with the highest average information gain. Two sets of analysis were performed to characterize the fully labeled data set. First, for each content category (ie, misinformation, fact, stigma, and positivity), the mean of each LIWC variable in the Reddit data was compared with each LIWC variable’s grand mean (ie, the weighted average of individual means). The LIWC grand mean was generated from the data used to create the LIWC software, which included 37,295 blog posts, 6179 pieces of expressive writing, 875 novels, 3232 transcripts of “natural speech,” 34,929 articles from the *New York Times,* and 35,269 Twitter posts [29]. Comparisons were made using Cohen *d*, which measures the standardized difference between 2 means. This standardized difference is considered “large” if above 0.8, "medium" if between 0.5 and 0.8, "small" if it was between 0.2 and 0.5, and "negligible" if below 0.2 [32]. This analysis was conducted to quantify the magnitude of the difference between the Reddit data and a set of heterogeneous texts, which could help identify uniquely enriched features in various types of obesity-related content on the platform.

In the second analysis, individual hurdle models were constructed for each variable derived from TF-IDF, VADER, and LIWC to evaluate the variations relative to each pair of labeled data. In the first step, a logistic regression model assessed the log-odds of obtaining a 0 result (eg, the log-odds of no first-person singular pronoun) for either misinformation (referenced against fact) or stigma (referenced against positivity). In the second step of the process, data were truncated at 0 before a standard linear regression model was constructed to model the difference in the mean value of each LIWC variable for either misinformation (referenced against fact) or stigma (referenced against positivity). In the standard linear regression model, this translates to the percentage of the sentence that comprises a given variable. For example, a β coefficient of 10 in the misinformation model for swear words implies that, on average, misinformation comments contained 10% more swear words (absolute difference) than factual content. This procedure was chosen given the 0-inflated, semicontinuous nature of the data and the ability to separately model the conditional presence or absence of a feature. This allows for the nature of the effects to vary; for example, a feature with a negative coefficient in the binary outcome model but a positive coefficient in the semicontinuous model suggests that the feature is rare but, when present, exists in large quantities [33,34]. This process was also repeated in a single model that compared each label category to the “Other” category as a reference. In both scenarios, log-odds and 95% CIs were used to report the findings from the logistic portion of the model, whereas β coefficients with 95% CIs were used to report the findings from the linear regression portion of the model. To account for false discovery, *P* values within this post hoc analysis were recomputed using a reformulation of the Benjamini-Hochberg procedure that adjusts the actual *P* values themselves while allowing for the false discovery rate (in this case, 5%) to parallel the traditional significance threshold [35,36]. *P* values were recomputed for each category comparison and for each step of the hurdle model (eg, logistic model for fact vs misinformation, continuous model for fact vs misinformation, and logistic model for stigma vs positivity) for a total of 570 tests. In a sensitivity analysis, this process was repeated only for the training data. In all cases, a significance threshold of 0.05 was used. Analyses were conducted in R (version 4.1.0) and Python (version 3.7.4) using packages such as *imblearn*, *nltk*, *sentence_transformers*, *sklearn*, and *xgboost* [37-41].

### Ethics Approval

No additional ethics approval was required for this study given the public nature of the relevant social media data [42]. The code is available at GitHub [43], while the data are available upon request.

## Results

### Data Description

The 764,179 sentences containing “obese” or “obesity” were derived from 689,447 top-level comments (1.11 sentences per comment on average) made between 2011 and 2019. Comments had an average score (ie, the difference between “upvotes” and “downvotes”) of 14.3 (SD 167). These comments were generated by a maximum of 375,053 unique authors (1.84 comments per author minimum), of which 22,418 (5.98%) were “deleted.” Of the 13,123 subreddits present within the data, the most frequent include r/AskReddit (n=97,540, a subreddit whereby users can “ask and answer thought-provoking questions”), r/fatlogic (n=77,417, a subreddit to “learn about or promote health eating habits, and dispel ‘fatlogic’ [ie, anything that deviates from the scientific facts of body weight management...]”), r/loseit (n=27,649, a subreddit “to discuss healthy and sustainable methods of weight loss”), r/fatpeoplehate (n=14,992, a now-banned subreddit that was dedicated to sharing derogatory content about individuals with obesity), and r/Fitness (n=13,107, a subreddit to discuss “physical fitness goals”).

### Model Selection

During the training process, XGBoost with oversampling achieved the best overall performance relative to all other models, including dummy classifiers (accuracy=0.69; weighted average precision=0.69; weighted average recall=0.69; weighted *F*_1_-score=0.63; Multimedia Appendix 4). XGBoost also performed the best under the original data conditions (accuracy=0.69; weighted average recall=0.69; weighted average precision=0.61; weighted *F*_1_-score=0.61), while naive Bayes performed best with the downsampled data (accuracy=0.30; weighted average precision=0.68; weighted average recall=0.30; weighted *F*_1_-score=0.25). All features were retained after performing forward variable selection, although the BERT-only model had identical accuracy, weighted average recall, and weighted average *F*_1_-score values compared with the full model; weighted average precision was 3 percentage points less (0.64 vs 0.69; Multimedia Appendix 5). Finally, the analysis of training size versus performance did not yield a plateau before the maximum value was reached, so all labeled posts were retained for training (Multimedia Appendix 6). The confusion matrix of the final XGBoost model with oversampling can be found in Multimedia Appendix 7**.**

### Model Implementation and Feature Importance

**Figure 2:**
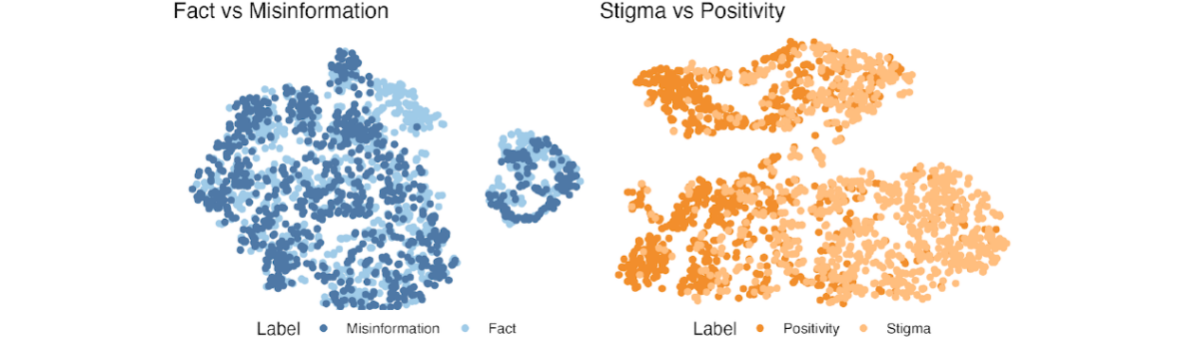
Visualization of each labeled category using t-distributed stochastic neighbor embedding (TSNE) dimensionality reduction. The left plot compares factual content and misinformation, while the right plot compares positivity and stigma. In both instances, the full set of labeled data was sampled randomly without replacement (points per category: n=1000).

After running the final XGBoost model with an oversampled training data set on the entire set of sentences, 3610 (0.47%) sentences were labeled as misinformation, 14,366 (1.88%) sentences were labeled as weight-related stigma, 14,799 (1.94%) were labeled as positivity, 68,276 (8.93%) were labeled as factual content, and the rest (663,128/764,179, 86.78%) were labeled as other. While misinformation and factual content appeared to cluster together in a 2D space (suggesting the presence of some similarity between the categories), stigma and positivity were more distinct (suggesting greater differentiation between the labeled categories; Figure 2).

There were 875 candidate features (11 from TF-IDF, 4 from VADER, 767 from BERT, and 93 from LIWC) for the model to evaluate. When frequency was used to assess which features contributed the most to the labeling process, the top 10 features were all dimensions of BERT, meaning that they did not have a direct interpretation. In contrast, 4 of the top-10 features with the highest information gain were non-BERT. These were first-person singular pronouns (eg, “I,” “me,” and “mine”), anger-tagged terms (eg, “hate,” “kill,” and “annoyed”), word count, and swear words. None of the BERT dimensions that contributed the most to information gain were in the top 10 of the frequent feature analysis.

### External Comparator Analysis With LIWC Grand Means

**Figure 3:**
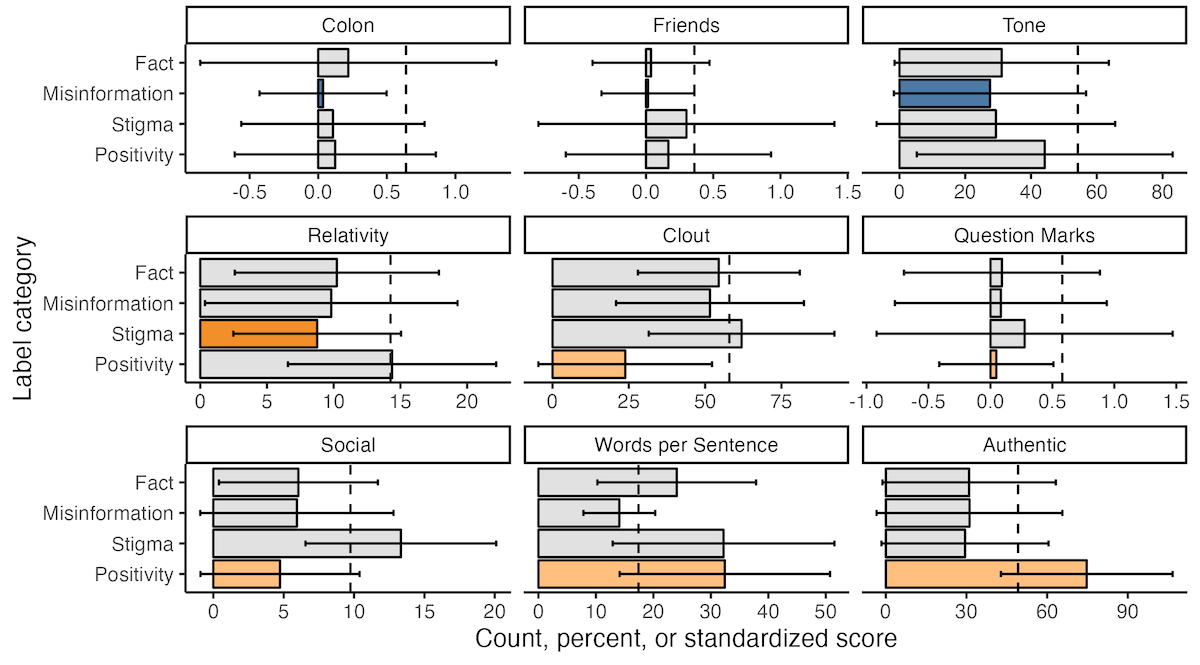
Comparisons between the grand mean of select psycholinguistic features from the 2015 Linguistic Inquiry and Word Count Program (LIWC 2015) and the 4 categories of interest: misinformation, factual content, stigma, and positivity. X-axis values for tone and clout are standardized scores based on the proprietary LIWC 2015 algorithm; x-axis for “Words per Sentence” is word count; x-axis for all other variables are the percentage of total words within the text. Only features that had 1 label category with a large, standardized difference (ie, Cohen d>0.80) are shown, with the substantially different category highlighted within each subplot. Error bars denote 1 SD above and below the mean, while the dashed lines denote the LIWC 2015 grand mean for each category. Note that x-axes are individualized per feature and cannot be compared across subplots. For a full list of numerical comparisons, see Multimedia Appendix 8.

Of the 93 LIWC features compared against the external grand mean (LIWC μ), 21 (23%) had a large, standardized difference for at least one (but not all) of the labels and 9 (10%) had a large, standardized difference for only 1 category (Figure 3). Compared with the LIWC texts, misinformation sentences had a lower average amount of friend-tagged terms (eg, “buddy” and “neighbor”; LIWC: μ=0.36, 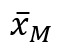
=0.01, s=0.35, Cohen *d*=1.30) and a more negative tone on average (LIWC: μ=54.22, 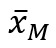
=27.5, s=29.2, Cohen *d*=0.92); however, this was not true of factual content. Sentences with stigma had a lower average amount of relativity-related terms (eg, “area,” “bend,” and “exit”) compared with the LIWC texts (LIWC: μ=14.26, 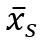
=8.76, s=6.28, Cohen *d*=0.87). Sentences tagged as positivity had lower clout on average (LIWC: μ=57.95, 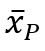
=23.8, s=28.4, Cohen *d*=1.20) and number of social-tagged terms (eg, “mate,” “talk,” “there,” and “child”; LIWC: μ=9.74, 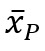
=4.74, s=5.66, Cohen *d*=0.88) but higher authenticity (LIWC: μ=49.17, 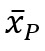
=74.7, s=31.9, Cohen *d*=0.80) relative to the LIWC Program texts. There were no terms with an exclusively large, standardized difference for facts, although both facts and positivity had fewer assent-tagged terms on average (eg, “agree,” “OK,” and “yes”) than the LIWC texts (LIWC: μ=0.95, 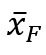
=0.09, s=0.75, Cohen *d*=1.14; 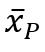
=0.18, s=0.84, Cohen *d*=0.92). A comparison of all LIWC features across the 4 categories of interest can be found in Multimedia Appendix 8.

### Feature Significance by Category

**Figure 4:**
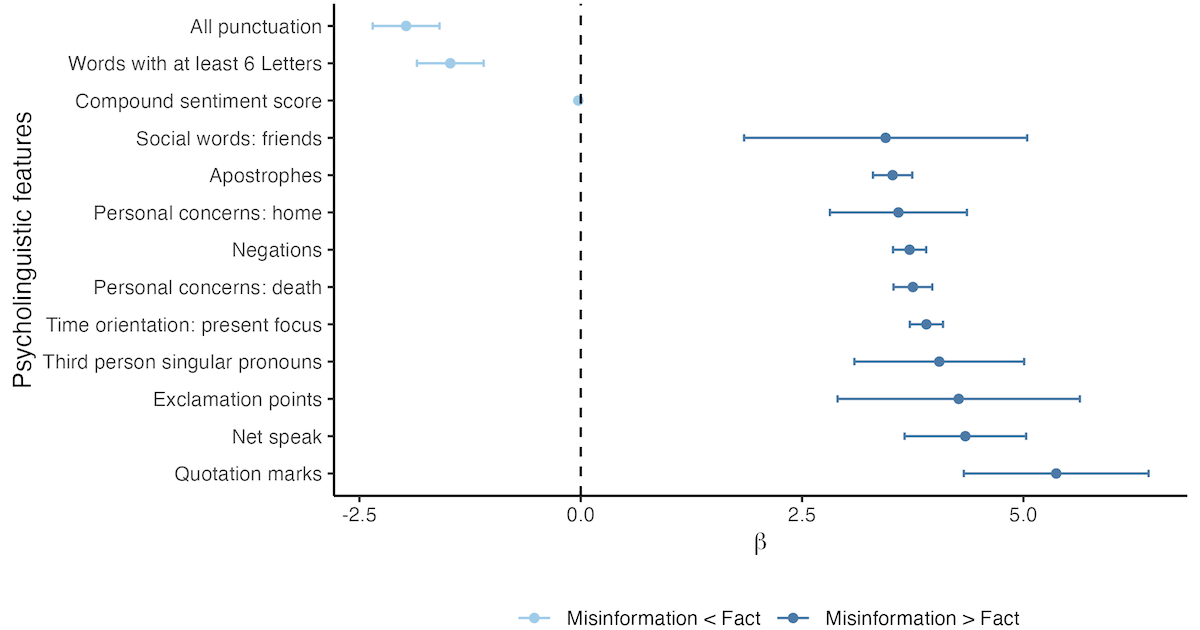
Select psycholinguistic features significantly different between fact and misinformation. Estimates are derived from the semicontinuous part of a 2-part hurdle model. The outcome of interest is the percentage of the sentence containing the psycholinguistic feature, and the exposure is the sentence label of either fact (reference, n=68,276) or misinformation (n=3610). Positive values suggest a higher prevalence of the feature among misinformation compared with fact, while negative values suggest a higher prevalence of the feature among fact compared with misinformation. Only the top 10 features with the largest effect size in each direction are shown, excluding 9 nonsignificant features and 74 significant features.

Many psycholinguistic features were significantly different between factual content and misinformation (Figure 4). For example, on average, there was a lower percentage of words of at least 6 letters in misinformation than fact (β=−1.47, 95% CI −1.85 to −1.10; *P*<.001); however, the log-odds of having no 6-letter words were not significantly different between categories (β=−0.03, 95% CI −0.43 to 0.32; *P*=.87). Misinformation also had a lower compound sentiment score (β=−0.03, 95% CI −0.04 to −0.01; *P*<.001), lower log-odds of containing no negation (eg, “no,” “not,” and “never”; β=−0.27, 95% CI −0.34 to −0.20; *P*<.001), and more negations overall (β=3.71, 95% CI 3.53-3.90; *P*<.001) compared with facts, suggesting a generally more negative and contrarian sentiment. Although misinformation also had a higher percentage of “net speak” on average (eg, “btw,” “lol,” and “thx”) compared with facts (β=4.34, 95% CI 3.66-5.03; *P*<.001), the presence of net speak within misinformation was not significantly different than the “Other” category (β=1.32, 95% CI −0.43 to 3.07, *P*=.15; see Multimedia Appendix 9 for a full list of comparisons between each label category and “Other”). Interestingly, misinformation also had higher log-odds of no net speak in any given sentence relative to factual content (β=1.69, 95% CI 1.37-2.05; *P*<.001) and other (β=2.17, 95% CI 1.85-2.53; *P*<.001). Taken together, this suggests that net speak is not always in misinformation but, when it is, it is present in large quantities. Although some differences in coefficient directionality emerged within the sensitivity analysis, they are likely attributable to the extremely small sample size of the data used for that analysis. A complete list of comparisons between factual content and misinformation, including findings from the logistic regression portion of the hurdle models, can be found in Multimedia Appendix 10.

**Figure 5:**
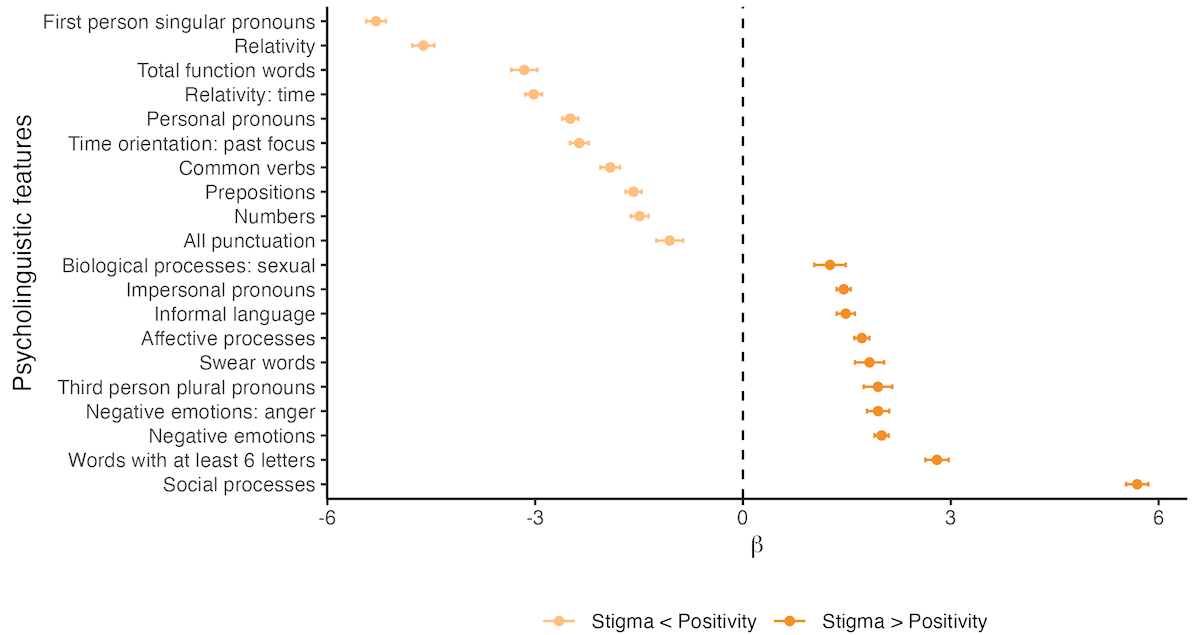
Select psycholinguistic features significantly different between positivity and stigma. Estimates are derived from the semicontinuous part of a 2-part hurdle model. The outcome of interest is the percentage of the sentence containing the psycholinguistic feature, and the exposure is the sentence label of either positivity (reference, n=14,799) or stigma (n=14,366). Positive values suggest a higher prevalence of the feature among stigma compared with positivity, while negative values suggest a higher prevalence of the feature among positivity compared with stigma. Only the top 10 features with the largest effect size in each direction are shown, excluding 20 nonsignificant features and 56 significant features.

Similarly, multiple psycholinguistic features were significantly different between positivity and stigma (Figure 5). There were significantly fewer references to all personal pronouns in sentences containing stigma compared with positivity, especially first-person singular personal pronouns (β=−5.30, 95% CI −5.44 to −5.16; *P*<.001). Stigma also had higher log-odds of containing no first-person singular pronouns relative to positivity (β=2.57, 95% CI 2.51-2.64; *P*=.15). In contrast, third-person plural pronouns were significantly more prevalent in sentences containing stigma (β=1.95, 95% CI 1.74-2.15; *P*<.001), and stigmatizing comments had lower log-odds of containing none of these pronouns relative to positivity pronouns (β=−2.12, 95% CI −2.19 to −2.05; *P*<.001). Negative emotions (β=2.00, 95% CI 1.90-2.10; *P*<.001) were also more prevalent in stigmatizing comments, which had lower log-odds of containing no words with a negative connotation relative to positivity (β=−0.90, 95% CI −0.95 to −0.85; *P*<.001). Similar to misinformation and factual content, some differences in directionality occurred within the sensitivity analysis, although this is likely attributable to variations in sample size. A complete list of comparisons, including log-odds for the first portion of the hurdle model, can be found in Multimedia Appendix 11.

## Discussion

### Principal Findings

This study is the first to comprehensively evaluate obesity-related content on Reddit. Using a multiclass XGBoost model and a suite of NLP features, misinformation was found to be relatively scarce on the platform. However, it had several unique features that distinguished it from both an external corpus and internal facts. There were a relatively similar number of sentences containing stigma and positivity, which could also be distinguished from one another. The novelty of the presented work is multifaceted and involves both methodological and applied contributions. From a methods standpoint, the pipeline developed here could be readily adapted to understand other public health topics on Reddit with minimum alterations required. From an applied standpoint, the findings provide a baseline for comparison in future work that may look at more specific facets of obesity content (such as nutrition, bariatric surgery, or antiobesity pharmacotherapies). Altogether, the results introduce a candidate set of features that could be explored as indicators for deleterious content, describe a framework for classifying obesity content, and provide important insights into the state of obesity content on the platform that could inform future health communication.

The underlying meanings of the psycholinguistic features enhanced by misinformation and stigma can help characterize how individuals on Reddit perceive obesity. For example, misinformation tended to have more of a present tense than factual content, a finding aligned with other studies on web-based misinformation [44]. Given that tense has been considered a proxy for “psychological distance” (whereby present and future tense suggest closer affinity compared with past tense), this may suggest that individuals experience stronger emotions when describing misinformation compared with factual content [30,45]. In addition, misinformation has a higher number of quotation marks and net speak relative to factual content. Although quotation marks are commonly used to signify an idea originally shared by someone other than the writer (such as the citation of an external reference), they can also be used as scare quotes to highlight something as ironic and distance the writer from the original meaning of the word [46]. Given that quotations could also be used when citing a formal manuscript, it is important to consider other markers beyond punctuation as possible indicators of obesity misinformation. For example, on average, net speak was present in higher quantities in misinformation than in facts. This suggests that individuals who describe untrue content may use more casual language, whereas those who describe something truthful may use more formal language. This could also indicate the presence of bots or trolls, who may use this kind of language to appear more human-like. However, the average amount of quotation marks and net speak within misinformation was not significantly different than the “other” category, which may mean that the labeled misinformation category is just capturing colloquial conversations and not actual misinformation.

In contrast, sentences containing stigma were enriched for social processes, negative emotions (including anger), and third-person plural pronouns (eg, “they”). This aligns with research within the vaccination space, which also found that stigmatized content was more negative and contained more third-person plural pronouns [47]. Similar to that study, this study found that positivity sentences contained a higher prevalence of first-person singular pronouns. Understanding the prevalence of pronouns within a text can help inform where a writer’s attention is placed; in this case, the enrichment of third-person pronouns in stigmatizing sentences may suggest that the stigmatizing language is directed at external individuals instead of the commenter [30]. Alternatively, these sentences could be made by users who are describing a situation in which they experienced stigma, as a prior study found that male students (who make up a majority of the Reddit userbase) tended to use more third-person pronouns when describing a scenario in which they were teased [48]. Although these smaller function words are typically removed during traditional NLP analysis, given their abundance in writing, the results presented here suggest that they may be important indicators that could inform how social media users feel about various topics.

The LIWC benchmark analysis demonstrated that each category of interest was also distinct from other heterogeneous texts. This may be due, in part, to the differences in who generated the content for each source. Although Reddit users are primarily men (61.8%) and aged ≤50 (58%) years, LIWC sources are heterogeneous [29]. Thus, this benchmark analysis may highlight unique dimensions of how the Reddit-specific demographic communicate about obesity. For example, there was no LIWC feature that was distinctly different between facts and the LIWC corpus. This may make sense, as facts are likely paraphrased or directly quoted from an external, reputable source. In contrast, stigma and positivity are likely original thoughts, explaining why they have some components that are “enriched” compared with the benchmarked texts. Misinformation may fall somewhere in between, as it could be either one’s own misconstrued idea about the topic or a quotation of a source that is misaligned with the scientific consensus. Future work could help inform the type of misinformation that is present on Reddit.

The classification pipeline developed here can be applied in many different ways. For example, tagging posts as misinformation or stigma could aid Reddit content moderators (ie, managers of subreddits that monitor content for policy violations) in identifying which comments may violate their community guidelines. Moderators can leverage historical data where they flagged and removed deleterious content as additional training data, creating a refined model that is tuned to the needs of each subreddit. Furthermore, researchers could use this model as a tool in a larger pipeline that seeks to understand the impact of user-facing flags on misinformation or stigma on behaviors. This may include behaviors of the initial commenter (eg, future comments containing misinformation or stigma) or other users (eg, percent of “upvotes,” number of response comments, or sentiment of responding comments). Automating the identification process of candidate comments would allow researchers to allocate more time to other areas of research, such as the development of specific countermessaging for each type of misinformation. Although the current classifier was developed specifically for obesity, it can be readily adapted to other topics of concern, especially if labeled training data already exist. Other adaptations could also include accounting and adjusting for common challenges in automated classification such as misspellings or sarcasm [49].

### Comparison With Prior Work

Prior text-based classification analysis on Reddit has been conducted for a myriad of health conditions. The bulk of this work has been focused on the mental health space, including the ability to classify posts into various mental illnesses or identify the risk of suicide [50,51]. Other studies have leveraged NLP tools to classify a user’s response to misinformation or to automate its detection [52,53]. Less work has been done within the stigma space, although some work exists that automates the detection of cyberbullying [54]. Many of these studies rely on similar features that are used in this work, most notably word embeddings [50,51,54]. Although these studies relied on context-independent models, such as Word2Vec or GLOVE, more recent work (especially within the COVID-19 space) has leveraged the bidirectionality of BERT to generate context-dependent embeddings [55,56]. The power of BERT was also evident in this study, as a majority of the most important model features were the dimensions of the BERT embeddings.

Although there are no prior studies that directly quantify obesity content on Reddit, the results presented here align in part with the findings on other social media platforms. A study that specifically looked at nutritional guidelines for selecting Facebook pages of bariatric surgery support groups found that over 50% of posts were either inaccurate or highly ambiguous [57]. This proportion is lower than the amount of inaccurate (ie, misinformation) or ambiguous (ie, other) information found in this study (87.2%), which could be explained by the difference in platform (ie, Reddit vs Facebook), topic (ie, obesity vs nutrition), or purpose (ie, specific advice seeking vs general content). In terms of stigma, a study on Twitter found that tweets on obesity often included jokes, and tweets containing derogatory jokes were retweeted more frequently than tweets with jokes that were not derogatory [11]. Although the presence of jokes was not assessed in this study, this may explain why third-person plural pronouns, informal speech, and negative tone were higher in sentences containing stigma compared with body positivity. This finding was verified in a separate Twitter study that also identified the presence of “unverified health content,” which may be considered a type of misinformation [13]. Altogether, although there may be common themes in communication that exist across platforms (such as the use of informal language and select function words and punctuation), there are also likely some platform-specific variations that warrant additional consideration. However, future work is required to precisely quantify the extent of variation in select linguistic and semantic features across platforms.

### Limitations

This study excels in its comprehensive approach in analyzing a large corpus of text that could be applied to health domains beyond obesity. Yet, there are several limitations that are important to note. Studies on social media are a crucial tool for understanding health attitudes and behaviors, but each platform has a different user base that may influence the study’s generalizability. This is particularly challenging to assess on Reddit, where users post anonymously. External surveys suggest that Reddit is predominately used by adult men, and thus, these results may not be generalizable to other populations or social media platforms. Despite this limitation, Reddit remains a vital platform to study given the growth of its userbase over the past decade (from an average of 46 million monthly active users in 2012 to 430 million monthly active users in 2019) and frequent usage among adults in the United States—approximately 18% report using Reddit “ever,” and of those that use Reddit, 43% rely on it for news [1,58,59]. Given the growth of the platform over the analyzed period, coupled with platform-specific changes (such as the ban of r/fatpeoplehate in 2015), it may also be important to explore temporal trends in obesity misinformation and stigma in future work. Second, this study relied on automated machine learning classifiers using their default hyperparameters to categorize each sentence, and as a result, there may have been misclassification of content. It is assumed that this would be randomly distributed and not disproportionately impact one class, but future work could improve the performance of the present model. This would also include refining the process such that fewer sentences received a label of “other,” tuning hyperparameters, including metadata in the classification algorithm (such as a post’s score or controversiality), leveraging stratified sampling when selecting promising keywords, and assessing the robustness of the model when novel types of misinformation and stigma are presented (eg, misinformation that is enriched for 6-letter words with less net speak).

Third, this study only focused on sentences containing “obese” or “obesity,” meaning that a large portion of content was excluded from analysis. These words were chosen given their direct relationship to the research question as compared with more colloquial terminology such as “weight” (which frequently referred to the heaviness of inanimate objects) and “fat” (which frequently referred to the dietary fat found in food). Although this resulted in high precision compared with the use of the full set of search terms, it likely decreased the recall of our study. In addition, given that the selected search terms could be considered clinical in nature, the true amount of misinformation and stigma on the platform is likely higher than what is presented here. Thus, future work should integrate this type of casual terminology into the data, starting with terms that are synonymous with obesity but do not have additional meanings (eg, “chubby”). In addition, this study does not consider any comment-level or user-level clustering, which can be addressed in future work. However, the impact on the current work is estimated to be minimum, as there were approximately 1.13 sentences per comment and a maximum of 1.83 comments per author (assuming all authors, including those with deleted account information, were distinct). Fourth, only top-level comments were analyzed, and future work could extend this to evaluate posts or lower-level comments. Fifth, the hurdle model point estimates in the labeled training data differed from the full model estimates on occasion. This may be attributable to the size of the training data, and future studies should explore this in detail. Finally, this study only focused on obesity, and future work could expand this to include tangential topics, such as weight loss, bariatric surgery, or nutrition.

### Conclusions

This study presents the first comprehensive analysis of the state of obesity-related misinformation, facts, stigma, and positivity on Reddit. Although the prevalence of misinformation and stigma appears low, these numbers are likely to be the lower bound of the actual amount on the platform. Given the rapid growth of the Reddit userbase, public health researchers should increasingly consider Reddit as a source of misinformation and stigma, especially because misinformation that begins on this platform could spread to other platforms and into everyday conversations. Furthermore, the identification of distinct psycholinguistic features that separate misinformation and stigma from facts and positivity can help Reddit moderators to more rapidly identify content that should be flagged and removed. Beyond this manual process, future work should consider how to leverage these features to aid in the automated identification of deleterious content on Reddit and other social media platforms in real time.

## Appendix

Multimedia Appendix 1 List of keywords used in Reddit search.

Multimedia Appendix 2 Confusion matrix for keyword refinement.

Multimedia Appendix 3 Comparison of sentiment and Linguistic Inquiry and Word Count output by whether a majority label was able to be reached among the three trained research assistants.

Multimedia Appendix 4 Model performance comparisons.

Multimedia Appendix 5 Model ablation testing results.

Multimedia Appendix 6 Training data size versus model performance.

Multimedia Appendix 7 Confusion matrix of final model.

Multimedia Appendix 8 Comparisons between Linguistic Inquiry and Word Count psycholinguistic features and label categories.

Multimedia Appendix 9 Full hurdle models: other versus all categories.

Multimedia Appendix 10 Full hurdle models: fact versus misinformation.

Multimedia Appendix 11 Full hurdle models: positivity versus stigma.

## Abbreviations

BERT: Bidirectional Encoder Representations from Transformers
LIWC: Linguistic Inquiry and Word Count program
NLP: natural language processing
RA: research assistant
TF-IDF: term frequency–inverse document frequency
VADER: Valence Aware Dictionary and Sentiment Reasoner
XGBoost: Extreme Gradient Boosting
