# Supplementary material for: Characterizing the Prevalence of Obesity Misinformation, Factual Content, Stigma, and Positivity on the Social Media Platform Reddit Between 2011 and 2019: Infodemiology Study
Source: J Med Internet Res. 2022 Dec 30;24(12):e36729. doi: 10.2196/36729 (PMC9840103; doi:10.2196/36729)
Supplement: Multimedia Appendix 1 [file jmir_v24i12e36729_app1.docx]

**Multimedia Appendix 1.** List of Keywords Used in Reddit Search

| **Formal Terminology** | **Synonymous/Colloquial Terminology** | **Nutrition and Fad Diets** |
| --- | --- | --- |
| - obesity  - obese  - appetite depressants  - reducing diet  - body weight  - lipectomy  - skinfold thickness  - anti obesity agents  - bariatrics  - overweight | - chubbiness  - fatness  - paunchiness  - plumpness  - rotundness  - stoutness  - chunkiness  - portliness  - fat  - adiposity  - corpulent  - adipose  - avoirdupois  - paunchy  - plump  - porcine  - portly  - pudgy  - rotund  - stout  - cellulite | - diet  - nutrition  - food fads  - nutritional science  - keto  - juice cleanse  - Nutrisystem  - paleo  - intermittent fasting  - flexitarian  - weight watchers  - weight  - pounds |
