# Supplementary material for: Characterizing the Prevalence of Obesity Misinformation, Factual Content, Stigma, and Positivity on the Social Media Platform Reddit Between 2011 and 2019: Infodemiology Study
Source: J Med Internet Res. 2022 Dec 30;24(12):e36729. doi: 10.2196/36729 (PMC9840103; doi:10.2196/36729)
Supplement: Multimedia Appendix 2 [file jmir_v24i12e36729_app2.docx]

**Multimedia Appendix 2: Keyword Refinement Process**

**Confusion matrix for keyword refinement.**

|  | Related to Obesity | Not Related to Obesity | Total |
| --- | --- | --- | --- |
| Would Have Been Captured by Keywords | 7^a^ | 8^b^ | 15 |
| Would Not Have Been Captured By Keywords | 7^c^ | 978^d^ | 985 |
| Total | 14 | 986 | 1000 |

a. Example of a sentence **related to obesity** that **includes a keyword** (**true positive**): “Remember that you didn’t get to this *weight* overnight.”

b. Example a sentence **not related to obesity** that **includes a keyword** (**false positive**): “I can tell you that on many light *weight* carbon frames you can actually deflect the top and down tubes just by squeezing them with thumb and index finger.”

c. Example of a sentence **related to obesity** that **does not include a keyword** (**false negative**): “Well you’re certainly a big boy, a large canvas so to speak.”

d. Example of a sentence **not related to obesity** that **does not include a keyword** (**true negative**): “I put it through google translate really quick.”

**Summary metrics before keyword refinement:**

**Accuracy:** $\frac{\boldsymbol{True Negatives+True Positives}}{\boldsymbol{Total}}\boldsymbol{=}\frac{\boldsymbol{978+7}}{\boldsymbol{978+7+7+8}}\boldsymbol{=}\frac{\boldsymbol{985}}{\boldsymbol{1000}}\boldsymbol{=99\%}$

**Precision/Positive Predictive Value:** $\frac{\boldsymbol{True Positives}}{\boldsymbol{True Positives+False Positives}}\boldsymbol{=}\frac{\boldsymbol{7}}{\boldsymbol{7+8}}\boldsymbol{=}\frac{\boldsymbol{7}}{\boldsymbol{15}}\boldsymbol{=47\%}$

**Negative Predictive Value:** $\frac{\boldsymbol{True Negatives}}{\boldsymbol{True Negatives+False Negatives}}\boldsymbol{=}\frac{\boldsymbol{978}}{\boldsymbol{978+7}}\boldsymbol{=}\frac{\boldsymbol{978}}{\boldsymbol{986}}\boldsymbol{=99\%}$

**Recall/Sensitivity:** $\frac{\boldsymbol{True Positives}}{\boldsymbol{True Positives+False Negatives}}\boldsymbol{=}\frac{\boldsymbol{7}}{\boldsymbol{7+7}}\boldsymbol{=}\frac{\boldsymbol{7}}{\boldsymbol{14}}\boldsymbol{=50\%}$

**Specificity:** $\frac{\boldsymbol{True Negatives}}{\boldsymbol{True Negatives+False Positives}}\boldsymbol{=}\frac{\boldsymbol{978}}{\boldsymbol{978+8}}\boldsymbol{=}\frac{\boldsymbol{978}}{\boldsymbol{986}}\boldsymbol{=99\%}$

**F1-Score:** $\frac{\boldsymbol{2*True Positives}}{\boldsymbol{2*True Positives+False Positives+False Negatives}}\boldsymbol{=}\frac{\boldsymbol{2*7}}{\boldsymbol{2*7+8+7}}\boldsymbol{=}\frac{\boldsymbol{14}}{\boldsymbol{29}}\boldsymbol{=48\%}$

**Confusion matrix for keyword refinement**

After the above analysis, keywords were restricted to just those sentences that mentioned “obese” or “obesity”

|  | Related to Obesity | Not Related to Obesity | Total |
| --- | --- | --- | --- |
| Would Have Been Captured by Keywords | 0 | 0 | 0 |
| Would Not Have Been Captured By Keywords | 14 | 986 | 1000 |
| Total | 14 | 986 | 1000 |

**Summary metrics after keyword refinement:**

**Accuracy:** $\frac{\boldsymbol{True Negatives+True Positives}}{\boldsymbol{Total}}\boldsymbol{=}\frac{\boldsymbol{986}}{\boldsymbol{978+7+7+8}}\boldsymbol{=}\frac{\boldsymbol{986}}{\boldsymbol{1000}}\boldsymbol{=99\%}$

**Precision/Positive Predictive Value:** $\frac{\boldsymbol{True Positives}}{\boldsymbol{True Positives+False Positives}}\boldsymbol{=0\%}$

**Negative Predictive Value:** $\frac{\boldsymbol{True Negatives}}{\boldsymbol{True Negatives+False Negatives}}\boldsymbol{=}\frac{\boldsymbol{986}}{\boldsymbol{986+14}}\boldsymbol{=}\frac{\boldsymbol{986}}{\boldsymbol{1,000}}\boldsymbol{=99\%}$

**Recall/Sensitivity:** $\frac{\boldsymbol{True Positives}}{\boldsymbol{True Positives+False Negatives}}\boldsymbol{=0\%}$

**Specificity:** $\frac{\boldsymbol{True Negatives}}{\boldsymbol{True Negatives+False Positives}}\boldsymbol{=}\frac{\boldsymbol{986}}{\boldsymbol{986}}\boldsymbol{=}\frac{\boldsymbol{986}}{\boldsymbol{986}}\boldsymbol{=100\%}$

**F1-Score:** $\frac{\boldsymbol{2*True Positives}}{\boldsymbol{2*True Positives+False Positives+False Negatives}}\boldsymbol{=0\%}$
