# Supplementary material for: Characterizing the Prevalence of Obesity Misinformation, Factual Content, Stigma, and Positivity on the Social Media Platform Reddit Between 2011 and 2019: Infodemiology Study
Source: J Med Internet Res. 2022 Dec 30;24(12):e36729. doi: 10.2196/36729 (PMC9840103; doi:10.2196/36729)
Supplement: Multimedia Appendix 3 [file jmir_v24i12e36729_app3.docx]

**Multimedia Appendix 3.** Category examples and comparison of sentiment and Linguistic Inquiry and Word Count output by whether a majority label was able to be reached among the three trained research assistants. Terms that had a median and IQR of 0 for both categories are excluded; all *P*-values are from a Wilcoxon signed rank test and are adjusted using the Benjamini-Hochberg procedure.

Verbatim Category Examples:

Misinformation: *The mentality of 'I was born this way, I can't help it' is a powerful causation of obesity in today's society.*

Fact: *Child obesity is a huge problem and there are so many factors, but I would say the majority of this is economic and/or education related - most parents do not feed their children oreos and pizza because they think it will help their children.*
Stigma: *morbid obesity.....just float around all day and cram groceries down your neck*

Positivity: ***Low muscle tone/obesity:** Strength comes in all shapes and forms.*

| Category | No Majority Label  n = 120  Median [IQR] | Majority Label  n = 1,871  Median [IQR] | *P*-value |
| --- | --- | --- | --- |
| Negative | 0.10 [0, 0.19] | 0.05 [0, 0.17] | .386 |
| Compound | 0 [-0.46, 0.24] | 0 [-0.36, 0.22] | .678 |
| Word Count | 20.0 [15.0, 25.0] | 17.0 [11.0, 26.0] | .325 |
| Analytical Thinking | 42.5 [14.5, 79.4] | 52.7 [14.6, 87.6] | .574 |
| Clout | 53.6 [17.0, 89.4] | 50.0 [20.2, 84.1] | .781 |
| Authenticity | 21.9 [1.74, 66.3] | 23.5 [1.79, 74.8] | .827 |
| Tone | 25.8 [1.00, 34.4] | 25.8 [1.00, 25.8] | .918 |
| Words per Sentence | 20.0 [15.0, 25.0] | 17.0 [11.0, 25.5] | .325 |
| Six Letter Words | 17.5 [11.8, 23.8] | 18.2 [11.1, 25.9] | .832 |
| Dictionary Words | 89.4 [83.3, 93.8] | 89.2 [81.8, 95.2] | .948 |
| Function Words | 52.0 [44.3, 57.1] | 50.0 [42.3, 57.1] | .678 |
| Pronouns | 12.5 [8.79, 16.7] | 12.0 [6.25, 18.2] | .828 |
| Personal | 7.85 [4.35, 12.0] | 7.14 [0, 12.5] | .668 |
| First Person Singular | 0 [0, 5.34] | 0 [0, 6.67] | .966 |
| Impersonal | 4.35 [0, 7.38] | 3.45 [0, 7.69] | .828 |
| **Articles** | 4.66 [0, 7.38] | 4.76 [0, 9.09] | .678 |
| Prepositions | 10.6 [7.14, 16.1] | 11.1 [6.67, 16.0] | .910 |
| Auxiliary Verbs | 10.0 [6.90, 14.3] | 10.0 [5.56, 14.3] | .729 |
| Common Adverbs | 4.88 [0, 8.70] | 4.35 [0, 8.33] | .770 |
| Conjunctions | 7.14 [3.81, 11.5] | 6.25 [0, 10.0] | .386 |
| Negations | 0 [0, 4.55] | 0 [0, 3.85] | .827 |
| Regular Verbs | 16.3 [11.8, 23.5] | 16.0 [10.5, 21.4] | .668 |
| Adjectives | 5.00 [0, 8.89] | 4.76 [0, 9.38] | .866 |
| Comparatives | 0 [0, 4.60] | 0 [0, 5.00] | .836 |
| Interrogatives | 0 [0, 2.29] | 0 [0, 1.45] | .910 |
| Quantifiers | 0 [0, 4.76] | 0 [0, 4.31] | .386 |
| Affect Words | 4.65 [0, 7.14] | 4 [0, 8.11] | .864 |
| Positive Emotion | 0 [0, 4.09] | 0 [0, 3.70] | .909 |
| Negative Emotion | 0 [0, 4.76] | 0 [0, 5.00] | .910 |
| Social Words | 8.70 [4.50, 14.3] | 8.70 [0, 14.8] | .857 |
| Cognitive Processes | 13.4 [7.14, 19.0] | 12.5 [5.88, 19.1] | .700 |
| Insight | 0 [0, 4.55] | 0 [0, 3.70] | .678 |
| Cause | 0 [0, 4.60] | 0 [0, 3.12] | .358 |
| Discrepancies | 0 [0, 4.35] | 0 [0, 2.41] | .793 |
| Tentativeness | 0 [0, 5.34] | 0 [0, 5.56] | .842 |
| Certainty | 0 [0, 3.30] | 0 [0, 0] | .827 |
| Differentiation | 3.12 [0, 7.28] | 2.13 [0, 7.14] | .827 |
| Perceptual Process | 0 [0, 4.09] | 0 [0, 3.70] | .759 |
| Biological Processes | 10.6 [7.55, 16.0] | 11.1 [7.41, 16.8] | .909 |
| Health | 7.69 [5.84, 12.5] | 8.33 [5.41, 13.3] | .793 |
| Ingesting | 8.00 [5.88, 11.8] | 8.33 [5.26, 12.5] | .948 |
| Core Drives and Needs | 4.45 [0, 8.33] | 4.76 [0, 9.09] | .857 |
| Power | 0 [0, 2.88] | 0 [0, 3.45] | .668 |
| Time Orientation – Past Focus | 0 [0, 4.76] | 0 [0, 4.76] | .973 |
| Time Orientation – Present Focus | 13.3 [7.92, 17.7] | 10.7 [5.56, 16.7] | .325 |
| Relativity | 8.22 [3.67, 14.3] | 9.09 [2.20, 15.0] | .793 |
| Space | 2.99 [0, 7.28] | 4.17 [0, 9.09] | .574 |
| Time | 0 [0, 5.19] | 0 [0, 6.25] | .889 |
| All Punctuation | 14.3 [8.99, 22.2] | 16.1 [10.0, 25.0] | .489 |
| Periods | 4.76 [3.49, 6.67] | 5.26 [3.03, 9.09] | .541 |
| Commas | 0 [0, 5.93] | 0 [0, 6.60] | .950 |
| Apostrophes | 0 [0, 5.07] | 0 [0, 5.00] | .814 |
