# Supplementary material for: Characterizing the Prevalence of Obesity Misinformation, Factual Content, Stigma, and Positivity on the Social Media Platform Reddit Between 2011 and 2019: Infodemiology Study
Source: J Med Internet Res. 2022 Dec 30;24(12):e36729. doi: 10.2196/36729 (PMC9840103; doi:10.2196/36729)
Supplement: Multimedia Appendix 4 [file jmir_v24i12e36729_app4.docx]

**Multimedia Appendix 4.** Model Performance Comparisons

| **Model Type** | **No Adjustment**  **N_train_ = 1,496** | **Oversampling**  **N_train_ = 5,115** | **Undersampling**  **N_train_ = 535** |
| --- | --- | --- | --- |
| Random Forest  Accuracy ^a^  Precision (Avg. ^b^ Weighted) ^c^  Recall (Avg. Weighted) ^d^  F1-Score (Avg. Weighted) ^e^ | **0.67**  **0.53**  **0.67**  **0.55** | **0.67**  **0.60**  **0.67**  **0.57** | **0.21**  **0.72**  **0.21**  0.10 |
| **XGBoost**  **Accuracy**  **Precision (Avg. Weighted)**  **Recall (Avg. Weighted)**  F1- Score (Avg. Weighted) | **0.69**  **0.61**  **0.69**  **0.61** | **0.69**  **0.69**  **0.69**  **0.63** | **0.28**  **0.65**  **0.28**  **0.24** |
| Support Vector Machine  Accuracy  Precision (Avg. Weighted)  Recall (Avg. Weighted)  F1- Score (Avg. Weighted) | 0.66  0.43  0.66  0.52 | **0.34**  **0.58**  **0.34**  **0.38** | 0.17  0.05  0.17  0.08 |
| Naïve Bayes  Accuracy  Precision (Avg. Weighted)  Recall (Avg. Weighted)  F1- Score (Avg. Weighted) | 0.30  **0.64**  0.30  0.32 | **0.29**  **0.65**  **0.29**  **0.31** | **0.30**  **0.68**  **0.30**  **0.31** |
| Multilayer Perceptron  Accuracy  Precision (Avg. Weighted)  Recall (Avg. Weighted)  F1- Score (Avg. Weighted) | 0.62  **0.58**  0.62  **0.59** | **0.59**  **0.59**  **0.59**  **0.59** | **0.27**  **0.61**  **0.27**  **0.25** |
| Dummy Classifier – Most Frequent  Accuracy  Precision (Avg. Weighted)  Recall (Avg. Weighted)  F1- Score (Avg. Weighted) | 0.66  0.43  0.66  0.52 | 0.13  0.02  0.13  0.02 | 0.13  0.02  0.13  0.02 |
| Dummy Classifier – Stratified  Accuracy  Precision (Avg. Weighted)  Recall (Avg. Weighted)  F1- Score (Avg. Weighted) | 0.46  0.44  0.46  0.45 | 0.18  0.45  0.18  0.23 | 0.17  0.50  0.17  0.18 |
| ^a^ Accuracy is defined as the proportion of all sentences that were correctly assigned their labeled class  ^c^ Avg: Average  ^d^ Precision (i.e., positive predictive value) is defined as the percent of sentences assigned a given label that are actually that label (e.g., the percent of all sentences labeled as misinformation that are actually misinformation). The average weighted precision is calculated as a weighted average of the precision values across all classes weighted by the number of sentences within that class  ^e^ Recall (i.e., sensitivity) is defined as the percent of all sentences of a given class that were correctly assigned their appropriate label (e.g., the percent of all misinformation sentences that were classified as misinformation). The average weighted recall is calculated as weighted average of the recall values across all classes weighted by the number of sentences within that class.  ^f^ F1-score is defined as the harmonic mean of precision and recall. The average weighted F1-score is calculated as a weighted average of the F1-scores across all classes weighted by the number of sentences within that class | | | |
