# Supplementary figures and images for: Characterizing the Prevalence of Obesity Misinformation, Factual Content, Stigma, and Positivity on the Social Media Platform Reddit Between 2011 and 2019: Infodemiology Study
Source: J Med Internet Res. 2022 Dec 30;24(12):e36729. doi: 10.2196/36729 (PMC9840103; doi:10.2196/36729)

**Multimedia Appendix 6.** Training Data Size vs. Model Performance


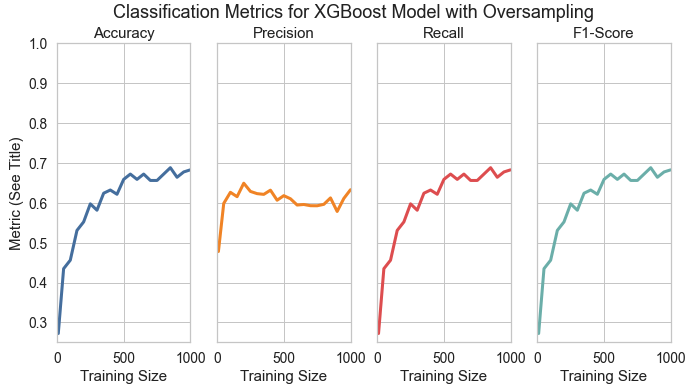

Supplement: Multimedia Appendix 6 [file jmir_v24i12e36729_app6.docx]
