# Supplementary material for: Characterizing the Prevalence of Obesity Misinformation, Factual Content, Stigma, and Positivity on the Social Media Platform Reddit Between 2011 and 2019: Infodemiology Study
Source: J Med Internet Res. 2022 Dec 30;24(12):e36729. doi: 10.2196/36729 (PMC9840103; doi:10.2196/36729)
Supplement: Multimedia Appendix 7 [file jmir_v24i12e36729_app7.docx]

**Multimedia Appendix 7:** Confusion Matrix of Final Model

|  | **Fact – M^a^** | **Misinformation - M** | **Stigma - M** | **Positivity - M** | **Other - M** | **Total** |
| --- | --- | --- | --- | --- | --- | --- |
| **Fact – RA^b^** | 18 | 1 | 0 | 1 | 28 | 48 |
| **Misinformation - RA** | 1 | 2 | 0 | 0 | 15 | 18 |
| **Stigma – RA** | 1 | 0 | 1 | 1 | 29 | 32 |
| **Positivity – RA** | 0 | 0 | 0 | 5 | 26 | 31 |
| **Other - RA** | 7 | 2 | 0 | 3 | 234 | 246 |
| **Total** | 27 | 5 | 1 | 10 | 332 | 375 |
| 1. Denotes label assigned by the model 2. Denotes label assigned by the research assistants | | | | | | |

| **Class** | **Accuracy** | **Precision**  **Positive Predictive Value** | **Negative Predictive Value** | **Recall**  **Sensitivity** | **Specificity** | **F1-Score** |
| --- | --- | --- | --- | --- | --- | --- |
| **Fact** | - | 66.6% | 89.0% | 37.5% | 96.4% | 48.0% |
| **Misinformation** | - | 40% | 94.2% | 11.1% | 98.9% | 17.4% |
| **Stigma** | - | 100% | 89.3% | 3.13% | 100% | 6.01% |
| **Positivity** | - | 50% | 90.7% | 16.1% | 96.6% | 24.4% |
| **Other** | - | 70.5% | 68.4% | 95.1% | 21.0% | 81.0% |
| **Average Weighted** | 69.3% | 69.4% | 75.9% | 69.3% | 47.4% | 67.3% |
