# Supplementary material for: Characterizing the Prevalence of Obesity Misinformation, Factual Content, Stigma, and Positivity on the Social Media Platform Reddit Between 2011 and 2019: Infodemiology Study
Source: J Med Internet Res. 2022 Dec 30;24(12):e36729. doi: 10.2196/36729 (PMC9840103; doi:10.2196/36729)
Supplement: Multimedia Appendix 8 [file jmir_v24i12e36729_app8.docx]

**Multimedia Appendix 8. Comparisons Between LIWC Psycholinguistic Features and Label Categories**

| **Table S1.** Select Comparisons in the Distribution of LIWC ^a^ Features by Label Category | | | | | | | | | |
| --- | --- | --- | --- | --- | --- | --- | --- | --- | --- |
| **LIWC Features** | **LIWC**  **Mean** | **Fact**  **Mean (SD ^b^)**  **N = 68,276** | **Cohen’s D ^c^** | **Misinformation**  **Mean (SD)**  **N = 3,610** | **Cohen’s D** | **Stigma**  **Mean (SD)**  **N = 14,366** | **Cohen’s D** | **Positivity**  **Mean (SD)**  **N = 14,799** | **Cohen’s D** |
| **Summary Variables**  Analytical Thinking  Clout  Authentic  Emotional Tone | 56.34  57.95  49.17  55.22 | 69.41 (29.73)  54.48 (26.52)  30.98 (32.23)  31.09 (32.57) | 0.44  0.13  0.56  0.71 | 64.68 (34.42)  51.61 (30.76)  31.13 (34.56)  27.52 (29.22) | 0.24  0.21  0.52  0.91 | 42.31 (30.97)  61.95 (30.42)  29.46 (31.06)  29.32 (36.25) | 0.45  0.13  0.63  0.69 | 39.35 (30.44)  23.83 (28.44)  74.73 (31.94)  44.16 (38.88) | 0.56  1.20  0.80  0.26 |
| **Language Metrics**  Words per Sentence  Words Greater than Six Letters  Dictionary Words | 17.40  15.60  85.18 | 24.07 (13.80)  26.44 (11.43)  85.12 (10.21) | 0.48  0.95  0.01 | 14.08 (6.25)  24.99 (11.2)  89.45 (9.37) | 0.53  0.84  0.46 | 32.20 (19.30)  17.08 (7.93)  90.47 (6.59) | 0.77  0.19  0.80 | 32.43 (18.30)  14.06 (7.18)  9.1.60 (6.27) | 0.82  0.21  1.02 |
| **Function Words**  Total Pronouns  Personal Pronouns  1^st^ Person Singular Pronouns  1^st^ Person Plural Pronouns  2^nd^ Person Pronouns  3^rd^ Person Singular Pronouns  3^rd^ Person Plural Pronouns  Impersonal Pronouns  Articles  Prepositions  Auxiliary Verbs  Common Adverbs  Conjunctions  Negations | 51.87  15.22  9.95  4.99  0.72  1.70  1.88  0.66  5.26  6.51  12.93  8.53  5.27  5.90  1.66 | 44.82 (10.41)  6.83 (5.92)  2.71 (4.00)  0.53 (1.87)  0.38 (1.49)  1.11 (2.98)  0.11 (0.88)  0.59 (1.81)  4.11 (4.50)  5.81 (4.83)  13.06 (6.06)  9.30 (5.72)  4.14 (4.70)  7.18 (5.19)  1.43 (2.84) | 0.68  1.42  1.81  2.39  0.23  0.20  2.02  0.04  0.25  0.14  0.02  0.13  0.24  0.25  0.08 | 48.08 (10.66)  7.70 (7.25)  3.19 (4.89)  0.66 (2.33)  1.05 (2.94)  0.81 (2.88)  0.12 (1.20)  0.56 (2.14)  4.51 (5.89)  8.22 (6.45)  11.95 (7.32)  11.91 (6.50)  3.75 (5.96)  5.20 (5.62)  2.94 (4.93) | 0.36  1.04  1.38  1.86  0.11  0.31  1.47  0.05  0.13  0.27  0.13  0.52  0.25  0.12  0.26 | 52.88 (8.81)  14.84 (6.60)  8.98 (5.51)  2.63 (3.93)  0.44 (1.70)  2.67 (4.58)  1.04 (2.83)  2.19 (3.41)  5.86 (4.73)  5.12 (3.93)  11.40 (5.41)  10.29 (5.47)  5.53 (4.60)  8.71 (4.86)  2.62 (3.09) | 0.11  0.06  0.18  0.60  0.16  0.21  0.30  0.45  0.13  0.35  0.28  0.32  0.06  0.58  0.31 | 56.05 (7.53)  16.17 (5.94)  12.31 (5.04)  10.30 (5.81)  0.13 (0.82)  1.25 (3.21)  0.37 (1.64)  0.26 (1.11)  3.85 (3.84)  4.46 (3.53)  13.29 (5.52)  11.20 (5.23)  6.40 (4.85)  8.96 (4.22)  2.18 (2.76) | 0.56  0.16  0.47  0.91  0.72  0.14  0.92  0.36  0.37  0.58  0.07  0.51  0.23  0.72  0.19 |
| **Grammar Other**  Regular Verbs  Adjectives  Comparatives  Interrogatives  Numbers  Quantifiers | 16.44  4.49  2.23  1.61  2.12  2.02 | 12.60 (6.77)  7.42 (6.56)  3.82 (4.96)  1.06 (2.23)  2.42 (5.03)  2.99 (3.92) | 0.57  0.45  0.32  0.25  0.06  0.25 | 15.97 (7.61)  5.82 (7.01)  3.00 (5.32)  1.03 (2.82)  1.79 (4.18)  2.97 (4.69) | 0.06  0.19  0.15  0.21  0.08  0.20 | 17.54 (6.34)  6.58 (5.36)  2.67 (3.45)  2.08 (2.73)  0.82 (1.85)  2.60 (3.15) | 0.17  0.39  0.13  0.17  0.70  0.18 | 19.53 (6.20)  6.85 (5.03)  3.37 (3.78)  1.18 (2.01)  2.11 (3.35)  2.40 (2.92) | 0.50  0.47  0.30  0.21  0  0.13 |
| **Affect Words**  Positive Emotion  Negative Emotion  Anxiety  Anger  Sadness | 5.57  3.67  1.84  0.31  0.54  0.41 | 4.68 (5.06)  1.83 (3.18)  2.81 (4.07)  0.67 (1.98)  0.25 (1.21)  0.78 (2.09) | 0.18  0.58  0.24  0.18  0.24  0.17 | 4.93 (6.17)  1.58 (3.68)  3.33 (5.17)  0.37 (1.70)  0.55 (2.15)  0.52 (2.01) | 0.10  0.57  0.29  0.03  0.01  0.05 | 8.05 (5.39)  2.99 (3.51)  5.00 (4.66)  0.39 (1.32)  2.57 (3.45)  0.57 (1.57) | 0.46  0.19  0.68  0.06  0.59  0.10 | 5.98 (4.44)  3.37 (3.72)  2.56 (2.98)  0.35 (1.13)  0.37 (1.20)  1.32 (2.16) | 0.09  0.08  0.24  0.03  0.14  0.42 |
| **Social Words**  Family  Friends  Female Referents  Male Referents | 9.74  0.44  0.36  0.98  1.65 | 6.05 (5.65)  0.20 (1.16)  0.04 (0.44)  0.37 (1.58)  0.22 (1.17) | 0.65  0.21  0.74  0.39  1.23 | 5.93 (6.87)  0.08 (0.85)  0.01 (0.35)  0.21 (1.55)  0.07 (0.78) | 0.55  0.42  1.00  0.50  2.03 | 13.33 (6.78)  0.32 (1.21)  0.30 (1.10)  1.57 (3.31)  0.62 (1.88) | 0.53  0.10  0.05  0.18  0.55 | 4.74 (5.66)  0.25 (1.02)  0.17 (0.76)  0.44 (1.73)  0.25 (1.17) | 0.88  0.19  0.25  0.31  1.19 |
| **Cognitive Processes**  Insight  Cause  Discrepancies  Tentativeness  Certainty  Differentiation | 10.61  2.16  1.40  1.44  2.52  1.35  2.99 | 13.08 (8.25)  2.47 (3.63)  2.72 (3.90)  1.46 (2.89)  3.25 (4.21)  1.15 (2.51)  4.08 (4.85) | 0.30  0.08  0.34  0.01  0.17  0.08  0.22 | 14.25 (9.65)  2.73 (4.32)  4.05 (5.63)  1.79 (3.78)  1.91 (3.90)  1.55 (3.53)  3.65 (5.52) | 0.38  0.13  0.47  0.09  0.16  0.06  0.12 | 13.73 (7.30)  2.19 (2.88)  2.13 (2.88)  1.76 (2.69)  3.35 (3.69)  2.01 (2.79)  4.42 (4.31) | 0.43  0.01  0.25  0.12  0.22  0.24  0.33 | 12.93 (7.18)  2.37 (2.91)  1.87 (2.69)  1.57 (2.53)  2.49 (3.09)  1.52 (2.38)  4.39 (4.19) | 0.32  0.07  0.17  0.05  0.01  0.07  0.33 |
| **Perpetual Processes**  Seeing  Hearing  Feeling | 2.70  1.08  0.83  0.64 | 1.74 (2.96)  0.46 (1.56)  0.18 (0.97)  0.77 (1.98) | 0.32  0.40  0.66  0.07 | 1.27 (3.19)  0.29 (1.49)  0.24 (1.42)  0.32 (1.56) | 0.45  0.53  0.42  0.21 | 2.36 (3.02)  0.95 (2.00)  0.53 (1.45)  0.66 (1.58) | 0.11  0.06  0.21  0.01 | 2.80 (3.09)  0.47 (1.32)  0.22 (0.86)  2.01 (2.64) | 0.03  0.46  0.71  0.52 |
| **Biological Processes**  Body  Health/Illness  Sexuality  Ingesting | 2.03  0.69  0.59  0.13  0.57 | 14.41 (7.73)  1.39 (2.96)  11.33 (6.63)  0.19 (1.12)  9.57 (5.73) | 1.60  0.24  1.62  0.05  1.57 | 15.96 (8.05)  0.76 (2.36)  13.19 (6.75)  0.05 (0.61)  11.87 (6.03) | 1.73  0.03  1.87  0.13  1.87 | 10.03 (5.77)  2.04 (2.93)  6.33 (3.95)  0.87 (2.14)  6.45 (4.04) | 1.39  0.46  1.46  0.35  1.45 | 9.57 (4.79)  1.01 (2.07)  6.63 (3.84)  0.13 (0.72)  7.13 (4.09) | 1.57  0.16  1.57  0  1.62 |
| **Core Drives and Needs**  Affiliation  Achievement  Power  Reward Focus  Risk/Prevention Focus | 6.93  2.05  1.30  2.35  1.46  0.47 | 6.51 (5.87)  0.80 (2.15)  1.32 (2.75)  2.96 (4.07)  0.78 (2.04)  1.61 (3.00) | 0.07  0.58  0.01  0.15  0.33  0.38 | 6.79 (7.19)  1.26 (3.18)  0.87 (2.59)  2.78 (4.70)  0.56 (2.12)  1.99 (4.02) | 0.02  0.25  0.17  0.09  0.43  0.38 | 6.20 (5.02)  1.17 (2.40)  1.36 (2.36)  2.35 (3.14)  1.29 (2.23)  0.66 (1.62) | 0.15  0.37  0.03  0  0.08  0.12 | 7.19 (4.92)  0.78 (1.90)  3.05 (3.35)  2.02 (2.76)  1.89 (2.68)  1.09 (1.99) | 0.05  0.67  0.52  0.12  0.16  0.31 |
| **Time Orientation**  Past Focus  Present Focus  Future Focus | 4.64  9.96  1.42 | 1.35 (2.87)  9.94 (6.03)  0.68 (1.88) |  | 0.87 (2.83)  14.15 (7.23)  0.50 (2.03) | 1.34  0.58  0.46 | 1.61 (2.90)  13.45 (6.16)  0.93 (1.93) | 1.04  0.57  0.26 | 4.88 (4.86)  12.62 (6.47)  0.95 (1.96) | 0.05  0.41  0.24 |
| **Relativity**  Motion  Space  Time | 14.26  2.15  6.89  5.46 | 10.23 (7.65)  1.38 (2.78)  5.94 (5.70)  2.97 (4.24) | 0.53  0.28  0.17  0.59 | 9.81 (9.46)  1.34 (3.27)  6.29 (7.48)  2.39 (4.54) | 0.47  0.25  0.08  0.68 | 8.76 (6.28)  1.15 (2.03)  4.59 (4.27)  3.13 (3.60) | 0.87  0.49  0.54  0.65 | 14.37 (7.80)  1.72 (2.52)  6.03 (4.76)  6.93 (5.58) | 0.01  0.17  0.18  0.26 |
| **Personal Concerns**  Work  Leisure  Home  Money  Religion  Death | 2.56  1.35  0.55  0.68  0.28  0.16 | 2.10 (3.45)  0.57 (1.90)  0.07 (0.63)  0.63 (2.09)  0.03 (0.48)  0.31 (1.35) | 0.13  0.41  0.76  0.02  0.51  0.11 | 1.21 (3.10)  0.40 (1.92)  0.08 (0.84)  0.85 (2.69)  0.06 (0.66)  1.70 (3.77) | 0.43  0.50  0.56  0.06  0.33  0.41 | 1.17 (2.35)  0.70 (1.73)  0.18 (0.82)  0.56 (1.67)  0.19 (0.97)  0.21 (0.95) | 0.59  0.38  0.46  0.07  0.09  0.05 | 1.38 (2.45)  0.81 (1.89)  0.11 (0.61)  0.20 (0.91)  0.06 (0.46)  0.06 (0.47) | 0.48  0.28  0.71  0.53  0.48  0.21 |
| **Informal Speech**  Swear Words  Netspeak  Assent  Nonfluencies  Fillers | 2.52  0.21  0.97  0.95  0.54  0.11 | 0.44 (1.51)  0.04 (0.48)  0.17 (0.87)  0.09 (0.75)  0.13 (0.84)  0.01 (0.25) | 1.37  0.35  0.92  1.14  0.49  0.40 | 0.43 (1.97)  0.12 (0.94)  0.07 (0.82)  0.14 (1.15)  0.08 (0.85)  0.01 (0.24) | 1.06  0.09  1.09  0.70  0.54  0.41 | 2.47 (3.52)  1.86 (3.13)  0.20 (0.88)  0.24 (1.04)  0.12 (0.66)  0.02 (0.32) | 0.01  0.53  0.88  0.68  0.65  0.27 | 0.77 (1.78)  0.21 (0.90)  0.21 (0.90)  0.18 (0.84)  0.14 (0.72)  0.02 (0.28) | 0.98  0  0.84  0.92  0.56  0.32 |
| **All Punctuation**  Periods  Commas  Colons  Semicolons  Question Marks  Exclamation Marks  Dashes  Quotation Marks  Apostrophes  Parentheses (Pairs)  Other Punctuation | 20.47  7.46  4.73  0.63  0.30  0.58  1.00  1.19  1.19  2.13  0.52  0.72 | 17.47 (11.37)  5.71 (3.77)  4.74 (5.52)  0.22 (1.08)  0.09 (0.69)  0.09 (0.79)  0.02 (0.39)  0.99 (2.81)  0.73 (2.86)  1.23 (2.79)  1.37 (3.53)  2.28 (5.24) | 0.34  0.47  0  0.39  0.30  0.62  2.50  0.07  0.33  0.44  0.24  0.30 | 15.47 (9.12)  8.49 (4.67)  2.75 (4.42)  0.04 (0.46)  0.03 (0.43)  0.08 (0.86)  0.07 (0.87)  0.29 (1.63)  0.42 (2.56)  2.54 (4.82)  0.21 (1.59)  0.55 (2.46) | 0.64  0.21  0.45  1.30  0.63  0.58  1.06  0.55  0.49  0.02  0.20  0.07 | 15.11 (8.86)  3.83 (3.21)  4.74 (5.24)  0.11 (0.67)  0.05 (0.45)  0.28 (1.19)  0.15 (0.99)  0.56 (1.78)  0.83 (2.52)  3.35 (3.90)  0.56 (1.89)  0.64 (2.17) | 0.70  1.14  0  0.80  0.55  0.25  0.85  0.35  0.33  0.23  0.02  0.04 | 16.24 (8.17)  3.90 (2.89)  4.55 (3.98)  0.12 (0.73)  0.07 (0.50)  0.05 (0.46)  0.28 (1.42)  0.55 (1.60)  0.66 (2.34)  3.90 (4.04)  1.43 (2.95)  0.74 (2.08) | 0.63  1.24  0.05  0.70  0.46  1.16  0.51  0.40  0.43  0.36  0.31  0 |
