# Supplementary material for: Characterizing the Prevalence of Obesity Misinformation, Factual Content, Stigma, and Positivity on the Social Media Platform Reddit Between 2011 and 2019: Infodemiology Study
Source: J Med Internet Res. 2022 Dec 30;24(12):e36729. doi: 10.2196/36729 (PMC9840103; doi:10.2196/36729)
Supplement: Multimedia Appendix 9 [file jmir_v24i12e36729_app9.docx]

**Multimedia Appendix 9. Full Hurdle Models: Other vs. All Categories**

|  | Semicontinuous Model | | Logistic Model | |
| --- | --- | --- | --- | --- |
| Outcome | β (95% CI^a^) | *P-*value^b^ | Log-Odds (95% CI) | *P-*value^c^ |
| TF-IDF^d^ |  |  |  |  |
| become obese  Fact  Misinformation  Positivity  Stigma | -0.01 (-0.01, 0)  -0.04 (-0.07, -0.01)  -0.01 (-0.02, -0.01)  0 (-0.01, 0.01) | .017  .009  .002  .575 | 0.30 (0.22, 0.38)  0.80 (0.39, 1.27)  -0.23 (-0.36, -0.09)  -0.12 (-0.26, 0.03) | < .001  < .001  .001  .118 |
| lose weight  Fact  Misinformation  Positivity  Stigma | -0.01 (-0.01, 0)  0 (-0.05, 0.05)  0.04 (0.03, 0.05)  -0.04 (-0.05, -0.02) | .118  .887  < .001  < .001 | -0.31 (-0.37, -0.26)  0.87 (0.50, 1.29)  -2.10 (-2.16, -2.05)  -0.31 (-0.42, -0.20) | < .001  < .001  < .001  < .001 |
| morbid obesity  Fact  Misinformation  Positivity  Stigma | 0 (0, 0.01)  0.01 (-0.01, 0.03)  -0.01 (-0.02, 0)  0 (-0.01, 0.02) | .295  .536  .058  .607 | -0.24 (-0.31, -0.18)  -0.13 (-0.41, 0.17)  0.01 (-0.14, 0.17)  0.26 (0.09, 0.44) | < .001  .405  .944  .004 |
| morbidly obese  Fact  Misinformation  Positivity  Stigma | 0.05 (0, 0.10)  0 (-0.01, 0.01)  -0.02 (-0.03, -0.01)  -0.02 (-0.03, -0.02) | .040  .979  < .001  < .001 | 1.97 (1.92, 2.03)  2.25 (1.98, 2.54)  0.20 (0.15, 0.26)  -0.78 (-0.82, -0.74) | < .001  < .001  < .001  < .001 |
| obese people  Fact  Misinformation  Positivity  Stigma | -0.02 (-0.03, -0.02)  0.03 (0, 0.05)  -0.07 (-0.09, -0.06)  -0.02 (-0.02, -0.01) | < .001  .052  < .001  < .001 | 0.16 (0.13, 0.20)  0.81 (0.62, 1.02)  1.12 (1.01, 1.23)  -0.68 (-0.73, -0.63) | < .001  < .001  < .001  < .001 |
| obese person  Fact  Misinformation  Positivity  Stigma | 0 (-0.01, 0.01)  -0.02 (-0.09, 0.04)  -0.03 (-0.04, -0.01)  -0.03 (-0.04, -0.02) | .974  .498  .001  < .001 | 0.66 (0.59, 0.73)  2.14 (1.57, 2.830  0.63 (0.48, 0.78)  -0.42 (-0.51, -0.33) | < .001  < .001  < .001  < .001 |
| obese woman  Fact  Misinformation  Positivity  Stigma | 0.02 (0, 0.04)  0.05 (-0.12, 0.22)  -0.02 (-0.05, 0.01)  -0.03 (-0.04, -0.02) | .043  .588  .228  < .001 | 2.63 (2.40, 2.88)  3.90 (2.41, 6.76)  1.67 (1.36, 2.00)  -0.15 (-0.28, -0.01) | < .001  < .001  < .001  .036 |
| obese women  Fact  Misinformation  Positivity  Stigma | -0.03 (-0.04, -0.02)  -0.05 (-0.16, 0.05)  -0.05 (-0.09, -0.01)  -0.01 (-0.02, 0) | < .001  .339  .018  .066 | 0.30 (0.22, 0.39)  2.67 (1.72, 4.06)  2.18 (1.78, 2.66)  -0.60 (-0.72, -0.49) | < .001  < .001  < .001  < .001 |
| obesity epidemic  Fact  Misinformation  Positivity  Stigma | 0 (-0.01, 0)  0.01 (0, 0.02)  -0.01 (-0.03, 0.01)  -0.02 (-0.03, 0) | .806  .063  .305  .054 | 0.04 (-0.02, 0.11)  -1.65 (-1.77, -1.53)  1.90 (1.59, 2.24)  1.14 (0.93, 1.38) | .218  < .001  < .001  < .001 |
| overweight obese  Fact  Misinformation  Positivity  Stigma | - 1. (0, 0.01)   2. (-0.01, 0.05)   -0.01 (-0.02, 0)  -0.04 (-0.05, -0.02) | .001  .201  .024  < .001 | -1.11 (-1.14, -1.08)  0.54 (0.29, 0.81)  -0.56 (-0.64, -0.48)  0.01 (-0.10, 0.11) | < .001  < .001  < .001  .948 |
| VADER^e^ |  |  |  |  |
| Negative Sentiment  Fact  Misinformation  Positivity  Stigma | -0.02 (-0.03, -0.02)  0.05 (0.04, 0.05)  -0.06 (-0.06, -0.06)  0 (-0.01, 0) | < .001  < .001  < .001  .001 | -0.48 (-0.46, -0.43)  -0.10 (-0.17, -0.04)  -0.80 (-0.84, -0.77)  -1.66 (-1.70, -1.61) | < .001  .002  < .001  < .001 |
| Positive Sentiment  Fact  Misinformation  Positivity  Stigma | -0.04 (-0.04, -0.04)  0.01 (0, 0.01)  -0.01 (-0.02, -0.01)  -0.04 (-0.04, -0.04) | < .001  .004  < .001  < .001 | -0.12 (-0.14, -0.11)  0.55 (0.48, 0.62)  -1.18 (-1.21, -1.14)  -1.03 (-1.07, -0.99) | < .001  < .001  < .001  < .001 |
| Compound Sentiment  Fact  Misinformation  Positivity  Stigma | -0.03 (-0.04, -0.03)  -0.06 (-0.08, -0.05)  0.10 (0.10, 0.11)  0.05 (0.04, 0.06) | < .001  < .001  < .001  < .001 | 0.05 (0.04, 0.07)  0.56 (0.48, 0.64)  -0.88 (-0.91, -0.85)  -0.06 (-0.09, -0.02) | < .001  < .001  < .001  .002 |
| LIWC^f^ |  |  |  |  |
| Language Metrics |  |  |  |  |
| Words Greater than Six Letters  Fact  Misinformation  Positivity  Stigma | 6.48 (6.39, 6.56)  5.00 (4.64, 5.36)  -5.66 (-5.84, -5.48)  -2.86 (-3.04, -2.67) | < .001  < .001  < .001  < .001 | -2.39 (-2.48, -2.31)  -2.43 (-2.81, -2.08)  -1.00 (-1.09, -0.91)  -1.83 (-1.97, -1.70) | < .001  < .001  < .001  < .001 |
| Function Words |  |  |  |  |
| All Pronouns  Fact  Misinformation  Positivity  Stigma | -5.68 (-5.75, -5.62)  -3.04 (-3.32, -2.75)  1.18 (1.06, 1.29)  0.23 (0.11, 0.35) | < .001  < .001  < .001  < .001 | 0.78 (0.76, 0.80)  1.19 (1.12, 1.26)  -4.30 (-4.65, -3.98)  -1.77 (-1.87, -1.67) | < .001  < .001  < .001  < .001 |
| Personal Pronouns  Fact  Misinformation  Positivity  Stigma | -4.76 (-4.83, -4.69)  -2.44 (-2.77, -2.11)  1.18 (1.08, 1.28)  -1.31 (-1.42, -1.21) | < .001  < .001  < .001  < .001 | 1.30 (1.28, 1.31)  1.53 (1.46, 1.60)  -3.69 (-3.87, -3.53)  -1.27 (-1.33, -1.21) | < .001  < .001  < .001  < .001 |
| First Person Singular Pronouns  Fact  Misinformation  Positivity  Stigma | -4.02 (-4.15, -3.88)  -1.71 (-2.33, -1.09)  2.11 (2.01, 2.20)  -3.19 (-3.33, -3.05) | < .001  < .001  < .001  < .001 | 1.78 (1.76, 1.81)  1.94 (1.83, 2.06)  -2.73 (-2.79, -2.67)  -0.15 (-0.19, -0.12) | < .001  < .001  < .001  < .001 |
| First Person Plural Pronouns  Fact  Misinformation  Positivity  Stigma | -1.67 (-1.78, -1.55)  1.41 (1.04, 1.78)  -2.83 (-3.18, -2.48)  -1.78 (-2.00, -1.56) | < .001  < .001  < .001  < .001 | 0.04 (0.01, 0.07)  -0.53 (-0.63, -0.44)  0.88 (0.79, 0.97)  -0.15 (-0.20, -0.09) | .007  < .001  < .001  < .001 |
| Second Person Pronouns  Fact  Misinformation  Positivity  Stigma | -2.00 (-2.11, -1.90)  -0.03 (-0.62, 0.57)  -2.22 (-2.43, -2.02)  -1.34 (-1.50, -1.19) | < .001  .941  < .001  < .001 | 0.20 (0.18, 0.22)  0.85 (0.74, 0.97)  0 (-0.04, 0.05)  -0.86 (-0.89, -0.82) | < .001  < .001  .883  < .001 |
| Third Person Singular Pronouns  Fact  Misinformation  Positivity  Stigma | -3.53 (-3.84, -3.23)  0.52 (-1.10, 2.13)  -3.47 (-3.82, -3.12)  -2.65 (-2.88, -2.42) | < .001  .556  < .001  < .001 | 2.12 (2.07, 2.18)  2.57 (2.30, 2.87)  0.85 (0.79, 0.92)  -0.16 ( -0.20, -0.11) | < .001  < .001  < .001  < .001 |
| Third Person Plural Pronouns  Fact  Misinformation  Positivity  Stigma | -1.56 (-1.64, -1.47)  0.75 (0.30, 1.19)  -2.73 (-2.96, -2.50)  -0.78 (-0.88, -0.68) | < .001  .001  < .001  < .001 | 0.20 (0.18, 0.22)  0.70 (0.58, 0.82)  0.79 (0.73, 0.85)  -1.33 (-1.37, -1.30) | < .001  < .001  < .001  < .001 |
| Impersonal Pronouns  Fact  Misinformation  Positivity  Stigma | -1.55 (-1.60, -1.50)  1.26 (1.02, 1.49)  -2.59 (-2.68, -2.49)  -1.13 (-1.22, -1.04) | < .001  < .001  < .001  < .001 | -0.16 (-0.17, -0.14)  0.37 (0.30, 0.43)  -0.40 (-0.44, -0.37)  -1.13 (-1.17, -1.09) | < .001  < .001  < .001  < .001 |
| Articles  Fact  Misinformation  Positivity  Stigma | -0.89 (-0.94, -0.85)  2.31 (2.14, 2.48)  -2.90 (-2.99, -2.82)  -2.27 (-2.35, -2.18) | < .001  < .001  < .001  < .001 | -0.46 (-0.48, -0.44)  -0.49 (-0.57, -0.42)  -0.57 (-0.60, -0.53)  -0.73 (-0.77, -0.69) | < .001  < .001  < .001  < .001 |
| Prepositions  Fact  Misinformation  Positivity  Stigma | 0.44 (0.39, 0.49)  0.70 (0.50, 0.90)  0.30 (0.20, 0.39)  -1.28 (-1.38, -1.19) | < .001  < .001  < .001  < .001 | -1.35 (-1.38, -1.31)  -0.20 (-0.29, -0.11)  -2.10 (-2.25, -2.00)  -1.31 (-1.38, -1.24) | < .001  < .001  < .001  < .001 |
| Auxiliary Verbs  Fact  Misinformation  Positivity  Stigma | -2.23 (-2.28, -2.18)  0.55 (0.33, 0.77)  -0.99 (-1.10, -0.89)  -1.68 (-1.79, -1.57) | < .001  < .001  < .001  < .001 | -0.65 (-0.68, -0.63)  -0.74 (-0.86, -0.62)  -2.21 (-2.32, -2.09)  -1.45 (-1.53, -1.37) | < .001  < .001  < .001  < .001 |
| Common Adverbs  Fact  Misinformation  Positivity  Stigma | -1.99 (-2.05, -1.93)  0.90 (0.60, 1.19)  -1.37 (-1.47, -1.27)  -1.98 (-2.08, -1.87) | < .001  < .001  < .001  < .001 | 0.05 (0.03, 0.06)  0.89 (0.82, 0.95)  -1.19 (-1.23, -1.15)  -0.87 (-0.91, -0.83) | < .001  < .001  < .001  < .001 |
| Conjunctions  Fact  Misinformation  Positivity  Stigma | -0.80 (-0.84, -0.75)  -0.09 (-0.30, 0.13)  -0.28 (-0.36, -0.20)  -0.21 (-0.30, -0.13) | < .001  .462  < .001  < .001 | -0.66 (-0.68, -0.64)  0.65 (0.59, 0.72)  -2.44 (-2.52, -2.35)  -1.75 (-1.82, -1.69) | < .001  < .001  < .001  < .001 |
| Negations  Fact  Misinformation  Positivity  Stigma | -1.79 (-1.86, -1.73)  1.92 (1.66, 2.18)  -2.67 (-2.78, -2.56)  -2.35 (-2.45, -2.25) | < .001  < .001  < .001  < .001 | 0.37 (0.35, 0.39)  0.10 (0.03, 0.17)  -0.60 (-0.64, -0.57)  -0.84 (-0.88, -0.81) | < .001  .006  < .001  < .001 |
| Other Grammar |  |  |  |  |
| Regular Verbs  Fact  Misinformation  Positivity  Stigma | -4.47 (-4.53, -4.40)  -1.27 (-1.53, -1.01)  1.84 (1.71, 1.96)  -0.08 (-0.21, 0.05) | < .001  < .001  < .001  .248 | -0.38 (-0.42, -0.35)  -0.93 (-1.14, -0.74)  -4.80 (-5.53, -4.21)  -2.74 (-2.99, -2.51) | < .001  < .001  < .001  < .001 |
| Adjectives  Fact  Misinformation  Positivity  Stigma | 0.42 (0.37, 0.47)  1.65 (1.39, 1.92)  -1.34 (-1.45, -1.24)  -1.30 (-1.41, -1.20) | < .001  < .001  < .001  < .001 | -0.75 (-0.77, -0.73)  0.27 (0.21, 0.34)  -1.36 (-1.40, -1.31)  -1.09 (-1.14, -1.05) | < .001  < .001  < .001  < .001 |
| Comparatives  Fact  Misinformation  Positivity  Stigma | 0.11 (0.06, 0.16)  2.30 (2.02, 2.58)  -1.71 (-1.81, -1.61)  -2.13 (-2.24, -2.02) | < .001  < .001  < .001  < .001 | -0.63 (-0.65, -0.62)  0.21 (0.14, 0.28)  -0.98 (-1.01, -0.95)  -0.63 (-0.67, -0.60) | < .001  < .001  < .001  < .001 |
| Interrogatives  Fact  Misinformation  Positivity  Stigma | -1.33 (-1.38, -1.27)  1.62 (1.31, 1.92)  -2.28 (-2.38, -2.19)  -1.53 (-1.62, -1.45) | < .001  < .001  < .001  < .001 | 0.20 (0.18, 0.22)  0.84 (0.74, 0.93)  -0.28 (-0.31, -0.24)  -0.91 (-0.94, -0.87) | < .001  < .001  < .001  < .001 |
| Numbers  Fact  Misinformation  Positivity  Stigma | 0.36 (0.27, 0.44)  1.11 (0.68, 1.54)  -2.53 (-2.68, -2.38)  -4.02(-4.23, -3.82) | < .001  < .001  < .001  < .001 | -0.32 (-0.34, -0.30)  0.20 (0.12, 0.28)  -0.78 (-0.82, -0.75)  0.09 (0.05, 0.13) | < .001  < .001  < .001  < .001 |
| Quantifiers  Fact  Misinformation  Positivity  Stigma | -0.36 (-0.41, -0.32)  1.95 (1.73, 2.17)  -1.92 (-2.01, -1.83)  -1.71 (-1.80, -1.62) | < .001  < .001  < .001  < .001 | -0.57 (-0.59, -0.56)  -0.02 (-0.09, 0.05)  -0.71 (-0.75, -0.68)  -0.79 (-0.82, -0.76) | < .001  .659  < .001  < .001 |
| Affect Words  Fact  Misinformation  Positivity  Stigma | -1.33 (-1.38, -1.27)  1.23 (0.98, 1.49)  -1.64 (-1.74, -1.54)  0.08 (-0.02, 0.17) | < .001  < .001  < .001  .129 | -0.21 (-0.23, -0.20)  0.32 (0.26, 0.39)  -1.28 (-1.32, -1.24)  -1.91 9-1.96, -1.86) | < .001  < .001  < .001  < .001 |
| Positive Emotion  Fact  Misinformation  Positivity  Stigma | -1.51 (-1.58, -1.45)  1.24 (0.90, 1.58)  -1.43 (-1.52, -1.33)  -1.71 (-1.81, -1.61) | < .001  < .001  < .001  < .001 | -0.02 (-0.04, 0)  0.73 (0.65, 0.81)  -1.14 (-1.18, -1.11)  -0.98 (-1.01, -0.94) | .017  < .001  < .001  < .001 |
| Negative Emotion  Fact  Misinformation  Positivity  Stigma | -0.92 (-0.98, -0.86)  1.84 (1.58, 2.10)  -2.57 (-2.67, -2.46)  -0.57 (-0.66, -0.47) | < .001  < .001  < .001  < .001 | -0.30 (-0.32, -0.28)  0.02 (-0.04, 0.09)  -0.71 (-0.74, -0.68)  -1.61 (-1.65, -1.57) | < .001  .520  < .001  < .001 |
| Anxiety  Fact  Misinformation  Positivity  Stigma | -0.22 (-0.29, -0.15)  1.99 (1.53, 2.45)  -1.85 (-2.01, -1.69)  -1.49 (-1.65, -1.33) | < .001  < .001  < .001  < .001 | -0.89 (-0.92, -0.87)  0.18 (0.04, 0.33)  -0.61 (-0.67, -0.56)  -0.63 (-0.68, -0.58) | < .001  .017  < .001  < .001 |
| Anger  Fact  Misinformation  Positivity  Stigma | -1.79 (-1.94, -1.63)  1.02 (0.47, 1.57)  -3.18 (-3.40, -2.96)  -1.23 (-1.34, -1.12) | < .001  < .001  < .001  < .001 | 1.03 (0.99, 1.06)  0.67 (0.55, 0.80)  0.17 (0.12, 0.22)  -1.85 (-1.88, -1.81) | < .001  < .001  < .001  < .001 |
| Sadness  Fact  Misinformation  Positivity  Stigma | -0.48 (-0.54, -0.41)  1.95 (1.55, 2.36)  -1.44 (-1.54, -1.35)  -1.63 (-1.77, -1.49) | < .001  < .001  < .001  < .001 | -0.59 (-0.61, -0.57)  0.32 (0.20, 0.45)  -1.59 (-1.63, -1.56)  -0.55 (-0.60, -0.50) | < .001  < .001  < .001  < .001 |
| Social Words  Fact  Misinformation  Positivity  Stigma | -4.51 (-4.58, -4.44)  -2.26 (-2.59, -1.94)  -5.07 (-5.22, -4.91)  0.62 (0.50, 0.75) | < .001  < .001  < .001  < .001 | 0.32 (0.30, 0.34)  0.97 (0.91, 1.04)  0.81 (0.78, 0.84)  -2.14 (-2.23, -2.05) | < .001  < .001  < .001  < .001 |
| Family  Fact  Misinformation  Positivity  Stigma | -1.30 (-1.47, -1.13)  0.80 (-0.52, 2.13)  -2.95 (-3.22, -2.68)  -2.68 (-2.93, -2.43) | < .001  .252  < .001  < .001 | 0.70 (0.66, 0.73)  1.95 (1.66, 2.27)  0.08 (0.02, 0.15)  -0.11 (-0.17, -0.05) | < .001  < .001  .011  < .001 |
| Friends  Fact  Misinformation  Positivity  Stigma | -1.85 (-2.16, -1.54)  1.59 (-1.32, 4.50)  -2.91 (-3.18, -2.64)  -2.50 (-2.72, -2.28) | < .001  .302  < .001  < .001 | 1.92 (1.84, 2.00)  3.49 (2.83, 4.34)  0.06 (-0.01, 0.13)  -0.44 (-0.49, -0.38) | < .001  < .001  .105  < .001 |
| Female Referents  Fact  Misinformation  Positivity  Stigma | -2.95 (-3.11, -2.80)  0.28 (-0.83, 1.39)  -3.44 (-3.73, -3.15)  -2.40 (-2.58, -2.23) | < .001  .640  < .001  < .001 | 0.95 (0.92, 0.99)  2.02 (1.82, 2.24)  0.67 (0.62, 0.73)  -0.63 (-0.66, -0.59) | < .001  < .001  < .001  < .001 |
| Male References  Fact  Misinformation  Positivity  Stigma | -3.19 (-3.38, -2.99)  -1.13 (-2.83, 0.57)  -4.27 (-4.61, -3.93)  -3.76 (-4.00, -3.53) | < .001  .210  < .001  < .001 | 1.28 (1.24, 1.31)  2.73 (2.42, 3.06)  0.85 (0.79, 0.92)  -0.02 (-0.06, 0.03) | < .001  < .001  < .001  .553 |
| Cognitive Processes  Fact  Misinformation  Positivity  Stigma | -1.10 (-1.17, -1.03)  1.32 (1.03, 1.62)  -1.76 (-1.90, -1.62)  -1.04 (-1.18, -0.90) | < .001  < .001  < .001  < .001 | -0.82 (-0.85, -0.80)  -0.22 (-0.31, -0.13)  -1.33 (-1.40, -1.26)  -1.50 (-1.58, -1.43) | < .001  < .001  < .001  < .001 |
| Insight  Fact  Misinformation  Positivity  Stigma | -0.67 (-0.72, -0.62)  1.61 (1.39, 1.83)  -1.93 (-2.02, -1.84)  -1.97 (-2.06, -1.87) | < .001  < .001  < .001  < .001 | -0.41 (-0.43, -0.40)  -0.04 (-0.11, 0.03)  -0.79 (-0.82, -0.76)  -0.65 (-0.69, -0.62) | < .001  .228  < .001  < .001 |
| Cause  Fact  Misinformation  Positivity  Stigma | -0.29 (-0.33, -0.24)  3.08 (2.88, 3.28)  -2.09 (-2.19, -1.99)  -1.88 (-1.98, -1.78) | < .001  < .001  < .001  < .001 | -0.65 (-0.66, -0.63)  -0.58 (-0.65, -0.52)  -0.59 (-0.63, -0.56)  -0.75 (-0.78, -0.71) | < .001  < .001  < .001  < .001 |
| Discrepancies  Fact  Misinformation  Positivity  Stigma | -1.10 (-1.16, -1.05)  1.59 (1.32, 1.87)  -2.26 (-2.37, -2.16)  -2.14 (-2.25, -2.04) | < .001  < .001  < .001  < .001 | -0.01 (-0.03, 0.01)  0.25 (0.17, 0.33)  -0.47 (-0.50, -0.43)  -0.61 (-0.64, -0.57) | .181  < .001  < .001  < .001 |
| Tentativeness  Fact  Misinformation  Positivity  Stigma | -0.96 (-1.02, -0.91)  0.58 (0.27, 0.89)  -2.67 (-2.78, -2.56)  -1.93 (-2.03, -1.83) | < .001  < .001  < .001  < .001 | -0.34 (-0.36, -0.33)  0.82 (0.75, 0.90)  -0.41 (-0.44, -0.38)  -0.76 (-0.80, -0.73) | < .001  < .001  < .001  < .001 |
| Certainty  Fact  Misinformation  Positivity  Stigma | -1.12 (-1.18, -1.06)  1.71 (1.43, 1.99)  -2.08 (-2.18, -1.98)  -1.69 (-1.78, -1.59) | < .001  < .001  < .001  < .001 | 0.07 (0.05, 0.08)  0.25 (0.17, 0.34)  -0.63 (-0.67, -0.60)  -0.95 (-0.99, -0.92) | < .001  < .001  < .001  < .001 |
| Differentiation  Fact  Misinformation  Positivity  Stigma | -1.41 (-1.47, -1.35)  0.95 (0.65, 1.25)  -2.48 (-2.59, -2.36)  -2.29 (-2.40, -2.18) | < .001  < .001  < .001  < .001 | -0.28 (-0.30, -0.27)  0.46 (0.39, 0.52)  -0.92 (-0.96, -0.89)  -0.85 (-0.88, -0.81) | < .001  < .001  < .001  < .001 |
| Perceptual Processes  Fact  Misinformation  Positivity  Stigma | -1.44 (-1.49, -1.38)  0.86 (0.54, 1.19)  -1.87 (-1.96, -1.78)  -1.95 (-2.05, -1.86) | < .001  < .001  < .001  < .001 | 0 (-0.02, 0.02)  0.90 (0.81, 0.99)  -1.03 (-1.07, -1.00)  -0.70 (-0.73, -0.67) | .977  < .001  < .001  < .001 |
| Seeing  Fact  Misinformation  Positivity  Stigma | -1.76 (-1.85, -1.66)  0.64 (0.01, 1.26)  -2.89 (-3.06, -2.72)  -2.32 (-2.45, -2.18) | < .001  .053  < .001  < .001 | 0.50 (0.47, 0.52)  1.45 (1.29, 1.61)  0.13 (0.08, 0.18)  -0.54 (-0.58, -0.50) | < .001  < .001  < .001  < .001 |
| Hearing  Fact  Misinformation  Positivity  Stigma | -1.20 (-1.32, -1.07)  1.84 (1.21, 2.47)  -2.43 (-2.64, -2.22)  -1.82 (-1.97, -1.66) | < .001  < .001  < .001  < .001 | 0.57 (0.53, 0.61)  0.86 (0.69, 1.05)  0.01 (-0.05, 0.07)  -0.77 (-0.82, -0.72) | < .001  < .001  .796  < .001 |
| Feeling  Fact  Misinformation  Positivity  Stigma | -0.72 (-0.78, -0.66)  1.23 (0.77, 1.70)  -1.02 (-1.09, -0.94)  -1.80 (-1.92, -1.68) | < .001  < .001  < .001  < .001 | -0.33 (-0.35, -0.31)  1.05 (0.90, 1.21)  -1.80 (-1.84, -1.77)  -0.47 (-0.51, -0.43) | < .001  < .001  < .001  < .001 |
| Body  Fact  Misinformation  Positivity  Stigma | -0.79 (-0.86, -0.72)  0.27 (-0.14, 0.67)  -2.54 (-2.67, -2.40)  -1.82 (-1.92, -1.71) | < .001  .214  < .001  < .001 | -0.17 (-0.19, -0.15)  0.76 (0.65, 0.86)  -0.24 (-0.28, -0.21)  -1.05 (-1.08, -1.02) | < .001  < .001  < .001  < .001 |
| Sexuality  Fact  Misinformation  Positivity  Stigma | -1.22 (-1.40, -1.04)  0.22 (-1.39, 1.83)  -3.11 (-3.47, -2.76)  -1.86 (-2.03, -1.69) | < .001  .801  < .001  < .001 | 0.48 (0.43, 0.52)  2.03 (1.68, 2.42)  0.36 (0.28, 0.45)  -1.38 (-1.43, -1.34) | < .001  < .001  < .001  < .001 |
| Core Drives and Needs  Fact  Misinformation  Positivity  Stigma | -0.68 (-0.73, -0.62)  1.61 (1.37, 1.85)  -1.36 (-1.46, -1.26)  -1.90 (-2.01, -1.80) | < .001  < .001  < .001  < .001 | -0.52 (-0.54, -0.51)  0.03 (-0.04, 0.10)  -1.45 (-1.49, -1.40)  -0.95 (-0.99, -0.91) | < .001  .405  < .001  < .001 |
| Affiliation  Fact  Misinformation  Positivity  Stigma | -1.66 (-1.75, -1.58)  1.26 (0.90, 1.62)  -2.69 (-2.84, -2.53)  -2.30 (-2.44, -2.17) | < .001  < .001  < .001  < .001 | 0.26 (0.24, 0.28)  0.28 (0.19, 0.37)  0 (-0.04, 0.04)  -0.40 (-0.44, -0.37) | < .001  < .001  .999  < .001 |
| Achievement  Fact  Misinformation  Positivity  Stigma | -0.58 (-0.64, -0.52)  1.31 (0.96, 1.65)  -0.77 (-0.85, -0.69)  -1.73 (-1.84, -1.63) | < .001  < .001  < .001  < .001 | -0.30 (-0.32, -0.28)  0.58 (0.48, 0.68)  -1.81 (-1.84, -1.77)  -0.70 (-0.73, -0.66) | < .001  < .001  < .001  < .001 |
| Power  Fact  Misinformation  Positivity  Stigma | -0.35 (-0.40, -0.30)  1.96 (1.72, 2.21)  -2.26 (-2.36, -2.15)  -1.85 (-1.95, -1.75) | < .001  < .001  < .001  < .001 | -0.60 (-0.62, -0.59)  0.01 (-0.06, 0.08)  -0.56 (-0.59, -0.53)  -0.68 (-0.71, -0.65) | < .001  .730  < .001  < .001 |
| Reward Focus  Fact  Misinformation  Positivity  Stigma | -0.98 (-1.05, -0.91)  1.31 (0.90, 1.72)  -1.30 (-1.39, -1.21)  -1.70 (-1.80, -1.60) | < .001  < .001  < .001  < .001 | 0.07 (0.05, 0.10)  0.91 (0.80, 1.04)  -1.27 (-1.31, -1.24)  -0.81 (-0.84, -0.77) | < .001  < .001  < .001  < .001 |
| Risk/Prevention Focus  Fact  Misinformation  Positivity  Stigma | -0.28 (-0.33, -0.22)  2.77 (2.52, 3.01)  -1.95 (-2.06, -1.84)  -2.12 (-2.25, -1.98) | < .001  < .001  < .001  < .001 | -0.86 (-0.87, -0.84)  -0.53 (-0.61, -0.45)  -0.82 (-0.86, -0.78)  -0.23 (-0.27, -0.19) | < .001  < .001  < .001  < .001 |
| Time Orientation |  |  |  |  |
| Past Focus  Fact  Misinformation  Positivity  Stigma | -2.99 (-3.07, -2.90)  -0.45 (-0.97, 0.06)  -1.19 (-1.30, -1.09)  -3.56 (-3.71, -3.41) | < .001  .090  < .001  < .001 | 0.44 (0.42, 0.45)  1.43 (1.33, 1.54)  -1.39 (-1.42, -1.35)  0.04 (0, 0.07) | < .001  < .001  < .001  .039 |
| Present Focus  Fact  Misinformation  Positivity  Stigma | -2.86 (-2.92, -2.80)  1.05 (0.81, 1.28)  -0.70 (-0.81, -0.58)  -0.04 (-0.16, 0.08) | < .001  < .001  < .001  .514 | -0.67 (0.69, -0.64)  -1.38 (-1.54, -1.23)  -1.63 (-1.71, -1.54)  -2.12 (-2.23, -2.01) | < .001  < .001  < .001  < .001 |
| Future Focus  Fact  Misinformation  Positivity  Stigma | -1.04 (-1.11, -0.97)  1.43 (1.00, 1.86)  -1.84 (-1.96, -1.73)  -1.88 (-2.00, -1.76) | < .001  < .001  < .001  < .001 | -0.05 (-0.07, -0.03)  0.77 (0.65, 0.90)  -0.72 (-0.76, -0.68)  -0.69 (-0.73, -0.65) | < .001  < .001  < .001  < .001 |
| Relativity  Fact  Misinformation  Positivity  Stigma | -1.19 (-1.26, -1.13)  1.04 (0.74, 1.34)  1.49 (1.37, 1.61)  -3.12 (-3.26, -2.99) | < .001  < .001  < .001  < .001 | -0.53 (-0.55, -0.51)  0.33 (0.26, 0.40)  -2.07 (-2.15, -1.99)  -0.62 (-0.67, -0.58) | < .001  < .001  < .001  < .001 |
| Motion  Fact  Misinformation  Positivity  Stigma | -0.49 (-0.55, -0.44)  2.00 (1.73, 2.27)  -1.52 (-1.61, -1.44)  -1.95 (-2.05, -1.85) | < .001  < .001  < .001  < .001 | -0.25 (-0.27, -0.23)  0.28 (0.20, 0.37)  -0.92 (-0.95, -0.89)  -0.46 (-0.49, -0.42) | < .001  < .001  < .001  < .001 |
| Space  Fact  Misinformation  Positivity  Stigma | -0.55 (-0.60, -0.50)  2.49 (2.25, 2.73)  -1.70 (-1.79, -1.60)  -2.59 (-2.70, -2.49) | < .001  < .001  < .001  < .001 | -0.49 (-0.50, -0.47)  0.15 (0.08, 0.21)  -1.11 (-1.15, -1.07)  -0.53 (-0.56, -0.49) | < .001  < .001  < .001  < .001 |
| Time  Fact  Misinformation  Positivity  Stigma | -1.60 (-1.66, -1.54)  0.47 (0.15, 0.79)  0.32 (0.22, 0.41)  -2.70 (-2.82, -2.59) | < .001  .005  < .001  < .001 | -0.04 (-0.05, -0.02)  0.73 (0.66, 0.80)  -1.73 (-1.77, -1.68)  -0.52 (-0.56, -0.49) | < .001  < .001  < .001  < .001 |
| Personal Concerns |  |  |  |  |
| Work  Fact  Misinformation  Positivity  Stigma | -0.43 (-0.49, -0.38)  1.35 (1.03, 1.67)  -2.03 (-2.15, -1.92)  -1.95 (-2.07, -1.82) | < .001  < .001  < .001  < .001 | -0.80 (-0.82, -0.79)  0.29 (0.20, 0.38)  -0.66 (-0.69, -0.62)  -0.39 (-0.43, -0.35) | < .001  < .001  < .001  < .001 |
| Leisure  Fact  Misinformation  Positivity  Stigma | -0.77 (-0.85, -0.68)  1.93 (1.39, 2.47)  -1.88 (-2.01, -1.75)  -2.03 (-2.17, -1.89) | < .001  < .001  < .001  < .001 | 0.13 (0.11, 0.16)  1.00 (0.85, 1.15)  -0.58 (-0.62, -0.54)  -0.45 (-0.49, -0.41) | < .001  < .001  < .001  < .001 |
| Home  Fact  Misinformation  Positivity  Stigma | -1.06 (-1.26, -0.87)  2.53 (1.44, 3.61)  -2.42 (-2.69, -2.15)  -2.13 (-2.36, -1.89) | < .001  < .001  < .001  < .001 | 0.88 (0.82, 0.94)  1.41 (1.11, 1.75)  0.02 (-0.06, 0.11)  -0.34 (-0.41, -0.27) | < .001  < .001  .628  < .001 |
| Money  Fact  Misinformation  Positivity  Stigma | -0.28 (-0.38, -0.19)  2.09 (1.71, 2.48)  -2.59 (-2.84, -2.34)  -1.80 (-1.97, -1.63) | < .001  < .001  < .001  < .001 | -0.47 (-0.49, -0.44)  -0.40 (-0.50, -0.29)  0.19 (0.12, 0.25)  -0.70 (-0.74, -0.65) | < .001  < .001  < .001  < .001 |
| Religion  Fact  Misinformation  Positivity  Stigma | -1.46 (-1.88, -1.03)  0.32 (-1.29, 1.92)  -3.19 (-3.74, -2.63)  -2.45 (-2.80, -2.10) | < .001  .712  < .001  < .001 | 1.12 (1.03, 1.21)  0.87 (0.55, 1.23)  0.12 (0.01, 0.24)  -0.90 (-0.97, -0.82) | < .001  < .001  .041  < .001 |
| Death  Fact  Misinformation  Positivity  Stigma | -1.67 (-1.80, -1.54)  2.08 (1.77, 2.40)  -3.64 (-4.11, -3.17)  -3.02 (-3.31, -2.73) | < .001  < .001  < .001  < .001 | -0.08 (-0.11, -0.04)  -1.35 (-1.43, -1.27)  1.07 (0.96, 1.18)  0 (-0.06, 0.07) | < .001  < .001  < .001  .929 |
| Informal Speech  Fact  Misinformation  Positivity  Stigma | -2.94 (-3.07, -2.81)  0.25 (-0.49, 0.98)  -3.45 (-3.66, -3.27)  -1.98 (-2.11, -1.85) | < .001  .538  < .001  < .001 | 0.74 (0.72, 0.77)  1.39 (1.25, 1.53)  -0.07 (-0.11, -0.03)  -1.31 (-1.34, -1.28) | < .001  < .001  < .001  < .001 |
| Swear Words  Fact  Misinformation  Positivity  Stigma | -2.23 (-2.60, -1.86)  -0.33 (-1.49, 0.83)  -3.47 (-3.77, -3.16)  -1.64 (-1.78, -1.50) | < .001  .600  < .001  < .001 | 2.09 (2.01, 2.16)  1.40 (1.18, 1.65)  0.10 (0.04, 0.16)  -2.02 (-2.06, -1.99) | < .001  < .001  .003  < .001 |
| Netspeak  Fact  Misinformation  Positivity  Stigma | -3.02 (-3.21, -2.84)  1.32 (-0.43, 3.07)  -3.39 (-3.72, -3.07)  -3.32 (-3.67, -2.98) | < .001  .152  < .001  < .001 | 0.48 (0.45, 0.52)  2.17 (1.85, 2.53)  0.13 (0.07, 0.20)  0.20 (0.14, 0.27) | < .001  < .001  < .001  < .001 |
| Assent  Fact  Misinformation  Positivity  Stigma | -1.78 (-2.05, -1.51)  0.94 (-0.21, 2.08)  -3.20 (-3.54, -2.86)  -2.89 (-3.20, -2.57) | < .001  .120  < .001  < .001 | 0.57 (0.52, 0.62)  0.60 (0.37, 0.84)  -0.49 (-0.56, -0.42)  -0.72 (-0.78, -0.65) | < .001  < .001  < .001  < .001 |
| Nonfluencies  Fact  Misinformation  Positivity  Stigma | -1.69 (-1.89 (-1.49)  1.53 (0.14, 2.92)  -2.87 (-3.21, -2.53)  -3.00 (-3.37, -2.63) | < .001  .035  < .001  < .001 | -0.08 (-0.13, -0.04)  0.97 (0.67, 1.30)  -0.48 (-0.56, -0.40)  -0.35 (-0.43, -0.26) | < .001  < .001  < .001  < .001 |
| Filler  Fact  Misinformation  Positivity  Stigma | -1.03 (-1.76, -0.31)  0.09 (-3.47, 3.65)  -2.69 (-3.54, -1.84)  -2.40 (-3.23, -1.56) | .006  .965  < .001  < .001 | 0.77 (0.61, 0.94)  1.07 (0.36, 1.99)  -0.43 (-0.61, -0.23)  -0.50 (-0.69, -0.31) | < .001  .010  < .001  < .001 |
| All Punctuation  Fact  Misinformation  Positivity  Stigma | -4.00 (-4.14, -3.86)  -5.97 (-6.55, -5.39)  -5.30 (-5.59, -5.01)  -6.36 (-6.65, -6.06) | < .001  < .001  < .001  < .001 | -1.63 (-1.73, -1.54)  -1.30 (-1.68, -0.97)  -2.82 (-3.22, -2.47)  -1.48 (-1.68, -1.29) | < .001  < .001  < .001  < .001 |
| Periods  Fact  Misinformation  Positivity  Stigma | -2.54 (-2.60, -2.47)  0.32 (0.04, 0.59)  -4.18 (-4.32, -4.04)  -4.03 (-4.18, -3.88) | < .001  .030  < .001  < .001 | -1.69 (-1.73, -1.65)  -1.83 (-2.01, -1.65)  -0.75 (-0.81, -0.69)  -0.30 (-0.35, -0.25) | < .001  < .001  < .001  < .001 |
| Commas  Fact  Misinformation  Positivity  Stigma | -0.75 (-0.81, -0.68)  -0.41 (-0.76, -0.05)  -2.34 (-2.46, -2.22)  -1.59 (-1.71, -1.46) | < .001  .028  < .001  < .001 | -0.56 (-0.58, -0.55)  0.55 (0.48, 0.62)  -1.14 (-1.18, -1.10)  -0.89 (-0.92, -0.85) | < .001  < .001  < .001  < .001 |
| Colons  Fact  Misinformation  Positivity  Stigma | -1.89 (-2.05, -1.73)  -0.45 (-2.36, 1.47)  -2.48 (-2.88, -2.08)  -2.60 (-3.00, -2.14) | < .001  .667  < .001  < .001 | -0.32 (-0.36, -0.29)  1.87 (1.49, 2.30)  0.10 (0.02, 0.19)  0.22 (0.13, 0.31) | < .001  < .001  .025  < .001 |
| Semicolons  Fact  Misinformation  Positivity  Stigma | -1.05 (-1.24, -0.85)  0.30 (-1.28, 1.87)  -1.85 (-2.25, -1.45)  -1.80 (-2.27, -1.34) | < .001  .728  < .001  < .001 | -0.56 (-0.61, -0.50)  0.88 (0.48, 1.34)  -0.50 (-0.61, -0.39)  -0.22 (-0.35, -0.09) | < .001  < .001  < .001  .001 |
| Question Marks  Fact  Misinformation  Positivity  Stigma | -3.90 (-4.34, -3.45)  -1.06 (-3.55, 1.44)  -5.74 (-6.89, -4.69)  -4.77 (-5.28, -4.26) | < .001  .430  < .001  < .001 | 1.64 (1.58, 1.70)  2.18 (1.88, 2.52)  1.86 (1.73, 2.00)  0.31 (0.25, 0.38) | < .001  < .001  < .001  < .001 |
| Exclamation Marks  Fact  Misinformation  Positivity  Stigma | -5.21 (-6.64, -3.77)  -0.94 (-5.49, 3.62)  -5.61 (-6.42, -4.79)  -6.00 (-7.06, -4.93) | < .001  .704  < .001  < .001 | 2.00 (1.88, 2.12)  1.38 (1.02, 1.79)  -0.74 (-0.81, -0.67)  -0.19 (-0.28, -0.10) | < .001  < .001  < .001  < .001 |
| Dashes  Fact  Misinformation  Positivity  Stigma | -3.16 (-3.36, -2.97)  -1.41 (-3.08, 0.26)  -4.97 (-5.41, -4.53)  -4.68 (-5.14, -4.23) | < .001  .109  < .001  < .001 | -0.56 (-0.59, -0.54)  1.08 (0.92, 1.25)  -0.33 (-0.38, -0.28)  -0.27 (-0.31, -0.22) | < .001  < .001  < .001  < .001 |
| Quotation Marks  Fact  Misinformation  Positivity  Stigma | -3.61 (-3.89, -3.37)  1.76 (0.04, 3.47)  -5.09 (-5.56, -4.61)  -5.07 (-5.50, -4.63) | < .001  .051  < .001  < .001 | 0 (-0.03, 0.02)  1.14 (0.96, 1.33)  -0.12 (-0.17, -0.07)  -0.38 (-0.43, -0.33) | .865  < .001  < .001  < .001 |
| Apostrophes  Fact  Misinformation  Positivity  Stigma | -2.80 (-2.90, -2.71)  0.72 (0.36, 1.08)  -2.35 (-2.47, -2.23)  -2.68 (-2.78, -2.53) | < .001  < .001  < .001  < .001 | 0.83 (0.81, 0.85)  0.52 (0.45, 0.60)  -1.07 (-1.10, -1.03)  -0.80 (-0.83, -0.77) | < .001  < .001  < .001  < .001 |
| Parentheses (Pairs)  Fact  Misinformation  Positivity  Stigma | -0.77 (-0.89, -0.65)  1.55 (0.14, 2.96)  -2.67 (-2.88, -2.46)  -3.42 (-3.73, -3.10) | < .001  .036  < .001  < .001 | -0.48 (-0.50, -0.46)  1.82 (1.60, 2.07)  -0.86 (-0.90, -0.82)  0.10 (0.04, 0.15) | < .001  < .001  < .001  .001 |
| Other Punctuation  Fact  Misinformation  Positivity  Stigma | -6.02 (-6.30, -5.73)  -5.93 (-8.26, -3.60)  -10.2 (-10.9, -9.50)  -9.74 (-10.6, -8.92) | < .001  < .001  < .001  < .001 | -0.69 (-0.71, -0.67)  0.98 (0.85, 1.12)  -0.09 (-0.13, -0.04)  0.20 (0.15, 0.25) | < .001  < .001  < .001  < .001 |
| ^a^ CI: Confidence Interval  ^b^ For all semicontinuous models, the predictor is a categorical variable that denotes the word label of either fact (reference) or misinformation. The outcome is the value associated with the psycholinguistic feature of interest, truncated at zero. *P-*values are adjusted based on the Benjamini Hochberg Procedure.  ^c^ For all logistic regression models, the predictor is a categorical variable that denotes the word label of either fact (reference) or misinformation. The outcome is a binary variable of either 0 (if the value for the psycholinguistic feature is above zero) or 1 (if the value of the psycholinguistic feature is zero). *P-*values are adjusted based on the Benjamini Hochberg Procedure.  ^d^ TF-IDF: Term Frequency – Inverse Document Frequency  ^e^ VADER: Valence Aware Dictionary and SEntiment Reasoner  ^f^ LIWC: Linguistic Inquiry and Word Count Program | | | | |
