# Supplementary material for: Characterizing the Prevalence of Obesity Misinformation, Factual Content, Stigma, and Positivity on the Social Media Platform Reddit Between 2011 and 2019: Infodemiology Study
Source: J Med Internet Res. 2022 Dec 30;24(12):e36729. doi: 10.2196/36729 (PMC9840103; doi:10.2196/36729)
Supplement: Multimedia Appendix 10 [file jmir_v24i12e36729_app10.docx]

**Multimedia Appendix 10. Full Hurdle Models: Fact vs. Misinformation**

|  | Full Data | | | | Labeled Data | | | |
| --- | --- | --- | --- | --- | --- | --- | --- | --- |
|  | Semicontinuous Model | | Logistic Model | | Semicontinuous Model | | Logistic Model | |
| Outcome | β (95% CI^a^) | *P-*value^b^ | Log-Odds (95% CI) | *P-*value^c^ | β (95% CI) | *P-*value^b^ | Log-Odds (95% CI) | *P-*value^c^ |
| VADER^e^ |  |  |  |  |  |  |  |  |
| Negative Sentiment | 0.07 (0.06, 0.07) | < .001 | 0.34 (0.28, 0.41) | < .001 | 0.06 (0.03, 0.09) | .004 | 0.09 (-0.42, 0.60) | .871 |
| Positive Sentiment | 0.05 (0.04, 0.05) | < .001 | 0.67 (0.60, 0.74) | < .001 | 0.02 (-0.02, 0.05) | .579 | 0.60 (0.08, 1.14) | .158 |
| Compound Sentiment | -0.03 (-0.04, -0.01) | .001 | 0.50 (0.42, 0.59) | < .001 | -0.02 (-0.13, 0.10) | .873 | 0.50 (-0.06, 1.09) | .348 |
| LIWC^f^ |  |  |  |  |  |  |  |  |
| Language Metrics |  |  |  |  |  |  |  |  |
| Words Greater than Six Letters | -1.47 (-1.85, -1.10) | < .001 | -0.03 (-0.43, 0.32) | .873 | -1.94 (-4.92, 1.05) | .427 | 0.39 (-1.18, 1.75) | .871 |
| Function Words | 3.24 (2.90, 3.59) | < .001 | -0.57 (-2.38, 0.59) | .473 | 1.82 (-0.98, 4.63) | .427 | 1.08 (-2.16, 4.32) | .754 |
| All Pronouns | 2.64 (2.44, 2.85) | < .001 | 0.41 (0.34, 0.48) | < .001 | 4.60 (2.97, 6.23) | < .001 | 0.10 (-0.50, 0.67) | .871 |
| Personal Pronouns | 2.32 (2.11, 2.52) | < .001 | 0.23 (0.16, 0.30) | < .001 | 3.60 (1.80, 5.39) | .003 | -0.12 (-0.63, 0.39) | .871 |
| First Person Singular Pronouns | 2.31 (1.94, 2.67) | < .001 | 0.16 (0.04, 0.28) | .010 | 1.38 (-0.74, 3.49) | .427 | -0.65 (-1.22, -0.06) | .158 |
| First Person Plural Pronouns | 3.07 (2.82, 3.33) | < .001 | -0.57 (-0.67, -0.47) | < .001 | 1.56 (-2.46, 5.58) | .621 | 0.03 (-0.82, 0.99) | .996 |
| Second Person Pronouns | 1.98 (1.54, 2.42) | < .001 | 0.65 (0.54, 0.77) | < .001 | 1.55 (-1.65, 4.75) | .561 | -0.07 (-0.74, 0.66) | .948 |
| Third Person Singular Pronouns | 4.05 (3.09, 5.01) | < .001 | 0.45 (0.17, 0.75) | .003 | 4.43 (0.25, 8.60) | .170 | -0.06 (-1.17, 1.24) | .995 |
| Third Person Plural Pronouns | 2.30 (2.00, 2.61) | < .001 | 0.50 (0.38, 0.63) | < .001 | 2.85 (-0.25, 5.94) | .222 | 0.13 (-0.71, 1.09) | .891 |
| Impersonal Pronouns | 2.81 (2.62, 2.99) | < .001 | 0.52 (0.45, 0.59) | < .001 | 2.48 (1.16, 3.79) | .004 | 0.16 (-0.35, 0.67) | .843 |
| Articles | 3.20 (3.05, 3.35) | < .001 | -0.03 (-0.10, 0.05) | .511 | 1.47 (0.14, 2.80) | .145 | 0.30 (-0.24, 0.83) | .576 |
| Prepositions | 0.26 (0.06, 0.45) | .011 | 1.15 (1.05, 1.25) | < .001 | -0.18 (-1.62, 1.26) | .881 | 0.86 (0.16, 1.54) | .158 |
| Auxiliary Verbs | 2.77 (2.59, 2.96) | < .001 | -0.08 (-0.21, 0.04) | .203 | 1.81 (0.25, 3.38) | .120 | 0.08 (-0.89, 0.95) | .948 |
| Common Adverbs | 2.89 (2.66, 3.11) | < .001 | 0.84 (0.77, 0.91) | < .001 | 0.76 (-1.29, 2 .82) | .636 | 0.59 (0.08, 1.11) | .158 |
| Conjunctions | 0.71 (0.51, 0.90) | < .001 | 1.31 (1.24, 1.38) | < .001 | -0.93 (-2.45, 0.60) | .466 | 0.59 (0.04, 1.13) | .180 |
| Negations | 3.71 (3.53, 3.90) | < .001 | -0.27 (-0.34, -0.20) | < .001 | 2.76 (1.10, 4.42) | .013 | -0.28 (-0.81, 0.26) | .616 |
| Other Grammar |  |  |  |  |  |  |  |  |
| Regular Verbs | 3.20 (2.98, 3.42) | < .001 | -0.55 (-0.76, -0.36) | < .001 | 4.70 (2.76, 6.63) | < .001 | -0.03 (-1.55, 1.21) | .996 |
| Adjectives | 1.23 (0.97, 1.50) | < .001 | 1.02 (0.95, 1.09) | < .001 | 1.39 (-0.87, 3.65) | .465 | 0.69 (0.17, 1.21) | .158 |
| Comparatives | 2.19 (1.92, 2.46) | < .001 | 0.84 (0.77, 0.91) | < .001 | 2.26 (-0.16, 4.68) | .220 | 0.50 (-0.02, 1.04) | .270 |
| Interrogatives | 2.94 (2.73, 3.15) | < .001 | 0.64 (0.54, 0.74) | < .001 | 0.40 (-1.19, 1.98) | .770 | 0.58 (-0.06, 1.27) | .348 |
| Numbers | 0.75 (0.30, 1.21) | .001 | 0.52 (0.44, 0.60) | < .001 | -0.92 (-5.43, 3.58) | .802 | 0.37 (-0.24, 1.01) | .575 |
| Quantifiers | 2.31 (2.12, 2.51) | < .001 | 0.56 (0.49, 0.63) | < .001 | 0.17 (-1.34, 1.69) | .881 | 0.11 (-0.39, 0.63) | .871 |
| Affect Words | 2.56 (2.35, 2.77) | < .001 | 0.54 (0.47, 0.60) | < .001 | 1.53 (0.09, 2.97) | .170 | 0.35 (-0.16, 0.86) | .482 |
| Positive Emotion | 2.75 (2.51, 3.00) | < .001 | 0.75 (0.66, 0.83) | < .001 | 0.34 (-1.39, 2.06) | .806 | 0.69 (0.11, 1.32) | .158 |
| Negative Emotion | 2.76 (2.54, 2.97) | < .001 | 0.32 (0.25, 0.39) | < .001 | 1.75 (0.37, 3.13) | .084 | -0.11 (-0.63, 0.41) | .871 |
| Anxiety | 2.21 (1.80, 2.62) | < .001 | 1.08 (0.93, 1.23) | < .001 | -0.03 (-2.42, 2.37) | .983 | 0.63 (-0.38, 1.88) | .576 |
| Anger | 2.81 (2.46, 3.16) | < .001 | -0.36 (-0.48, -0.23) | < .001 | 2.67 (-0.15, 5.48) | .214 | -1.29 (-2.08, -0.51) | .103 |
| Sadness | 2.43 (2.08, 2.77) | < .001 | 0.91 (0.79, 1.04) | < .001 | 0.89 (-1.07, 2.86) | .579 | 0.18 (-0.66, 1.13) | .871 |
| Social Words | 2.25 (2.03, 2.47) | < .001 | 0.66 (0.59, 0.72) | < .001 | 2.31 (0.55, 4.06) | .071 | -0.22 (-0.80, 0.33) | .754 |
| Family | 2.11 (1.21, 3.00) | < .001 | 1.25 (0.96, 1.57) | < .001 | 1.90 (-2.79, 6.58) | .596 | 0.40 (-0.63, 1.67) | .789 |
| Friends | 3.44 (1.84, 5.04) | < .001 | 1.58 (0.91, 2.43) | < .001 | -1.44 (-10.5, 7.65) | .802 | 0.55 (-1.30, 3.51) | .871 |
| Female Referents | 3.23 (2.59, 3.88) | < .001 | 1.07 (0.86, 1.29) | < .001 | 7.61 (1.48, 13.7) | .095 | 0.22 (-0.73, 1.36) | .871 |
| Male References | 2.06 (1.16, 2.96) | < .001 | 1.45 (1.14, 1.79) | < .001 | 3.33 (-0.61, 7.28) | .253 | 0.63 (-0.50, 2.11) | .635 |
| Cognitive Processes | 2.42 (2.15, 2.70) | < .001 | 0.61 (0.51, 0.70) | < .001 | 2.26 (0.07, 4.45) | .179 | -0.35 (-1.24, 0.42) | .713 |
| Insight | 2.28 (2.09, 2.48) | < .001 | 0.37 (0.30, 0.44) | < .001 | 1.30 (-0.06, 2.66) | .214 | -0.40 (-0.91, 0.12) | .415 |
| Cause | 3.36 (3.17, 3.55) | < .001 | 0.06 (-0.01, 0.13) | .089 | 2.51 (0.88, 4.13) | .023 | -0.10 (-0.61, 0.42) | .871 |
| Discrepancies | 2.70 (2.48, 2.93) | < .001 | 0.27 (0.19, 0.35) | < .001 | 1.75 (0.01, 3.49) | .183 | -0.25 (-0.81, 0.33) | .713 |
| Tentativeness | 1.54 (1.29, 1.80) | < .001 | 1.17 (1.09, 1.25) | < .001 | 0.09 (-1.50, 1.68) | .930 | 0.51 (0, 1.04) | .246 |
| Certainty | 2.83 (2.61, 3.04) | < .001 | 0.19 (0.10, 0.27) | < .001 | 0.84 (-1.13, 2.81) | .595 | 0.38 (-0.21, 1.01) | .537 |
| Differentiation | 2.36 (2.13, 2.60) | < .001 | 0.74 (0.67, 0.81) | < .001 | 1.07 (-0.49, 2.63) | .414 | 0.12 (-0.39, 0.63) | .871 |
| Perceptual Processes | 2.30 (2.07, 2.53) | < .001 | 0.90 (0.81, 0.99) | < .001 | 0.76 (-1.26, 2.78) | .636 | 0.71 (0.14, 1.32) | .158 |
| Seeing | 2.39 (2.00, 2.78) | < .001 | 0.95 (0.79, 1.12) | < .001 | 0.67 (-1.43, 2.76) | .684 | 0.14 (-0.75, 1.18) | .891 |
| Hearing | 3.03 (2.62, 3.45) | < .001 | 0.29 (0.11, 0.48) | .002 | 1.92 (-2.40, 6.23) | .582 | 0.44 (-0.49, 1.56) | .713 |
| Feeling | 1.96 (1.59, 2.32) | < .001 | 1.38 (1.23, 1.54) | < .001 | -0.31 (-4.61, 3.99) | .919 | 1.16 (0.28, 2.25) | .158 |
| Biological Processes |  |  |  |  |  |  |  |  |
| Body | 1.06 (0.72, 1.39) | < .001 | 0.93 (0.82, 1.03) | < .001 | -1.24 (-3.42, 0.95) | .489 | 0.36 (-0.28, 1.07) | .608 |
| Sexuality | 1.44 (0.34, 2.54) | .012 | 1.55 (1.21, 1.94) | < .001 | 1.53 (-13.7, 16.8) | .881 | 1.36 (-0.30, 4.27) | .482 |
| Core Drives and Needs | 2.29 (2.07, 2.50) | < .001 | 0.55 (0.49, 0.62) | < .001 | 0.94 (-0.98, 2.86) | .561 | 0.66 (0.15, 1.18) | .158 |
| Affiliation | 2.92 (2.68, 3.17) | < .001 | 0.02 (-0.07, 0.11) | .726 | 1.07 (-1.43, 3.56) | .595 | 0.12 (-0.54, 0.83) | .871 |
| Achievement | 1.89 (1.59, 2.18) | < .001 | 0.88 (0.78, 0.98) | < .001 | -0.05 (-1.67, 1.57) | .963 | 0.30 (-0.37, 1.03) | .713 |
| Power | 2.31 (2.09, 2.53) | < .001 | 0.62 (0.54, 0.69) | < .001 | 0.96 (-0.88, 2.80) | .543 | 0.68 (0.12, 1.28) | .158 |
| Reward Focus | 2.29 (1.98, 2.60) | < .001 | 0.84 (0.72, 0.96) | < .001 | 1.56 (-0.18, 3.30) | .227 | 0.17 (-0.55, 0.98) | .871 |
| Risk/Prevention Focus | 3.04 (2.83, 3.26) | < .001 | 0.32 (0.24, 0.40) | < .001 | 1.46 (-0.14, 3.06) | .223 | 0.41 (-0.19, 1.05) | .482 |
| Time Orientation |  |  |  |  |  |  |  |  |
| Past Focus | 2.53 (2.20, 2.86) | < .001 | 0.99 (0.89, 1.10) | < .001 | 2.96 (0.54, 5.38) | .095 | 0.31 (-0.28, 0.94) | .632 |
| Present Focus | 3.90 (3.72, 4.09) | < .001 | -0.71 (-0.87, -0.56) | < .001 | 3.08 (1.26, 4.90) | .013 | -0.20 (-1.23, 0.69) | .871 |
| Future Focus | 2.47 (2.15, 2.78) | < .001 | 0.83 (0.70, 0.96) | < .001 | 1.48 (-1.03, 3.98) | .471 | 0.29 (-0.45, 1.13) | .765 |
| Relativity | 2.23 (1.96, 2.51) | < .001 | 0.86 (0.79, 0.93) | < .001 | -0.99 (-3.01, 1.02) | .561 | 0.48 (-0.10, 1.05) | .351 |
| Motion | 2.49 (2.25, 2.74) | < .001 | 0.53 (0.45, 0.62) | < .001 | 0.49 (-1.31, 2.29) | .743 | 0.44 (-0.18, 1.12) | .482 |
| Space | 3.04 (2.81, 3.26) | < .001 | 0.63 (0.57, 0.70) | < .001 | 0.30 (-1.30, 1.91) | .811 | 0.44 (-0.07, 0.95) | .349 |
| Time | 2.07 (1.82, 2.33) | < .001 | 0.77 (0.69, 0.84) | < .001 | 0.32 (-1.62, 2.26) | .837 | 0.40 (-0.12, 0.94) | .431 |
| Personal Concerns |  |  |  |  |  |  |  |  |
| Work | 1.78 (1.50, 2.06) | < .001 | 1.09 (1.00, 1.18) | < .001 | 0.56 (-1.34, 2.46) | .717 | 0.69 (0.04, 1.41) | .219 |
| Leisure | 2.70 (2.25, 3.15) | < .001 | 0.86 (0.72, 1.02) | < .001 | 1.15 (-1.59, 3.89) | .595 | -0.02 (-0.83, 0.89) | .996 |
| Home | 3.59 (2.81, 4.36) | < .001 | 0.53 (0.22, 0.87) | .002 | - | - | - | - |
| Money | 2.38 (2.03, 2.72) | < .001 | 0.07 (-0.03, 0.18) | .216 | -1.03 (-4.17, 2.12) | .684 | 0.22 (-0.61, 1.17) | .871 |
| Religion | 1.78 (0.70, 2.85) | < .001 | -0.24 (-0.58, 0.12) | .199 | - | - | - | .- |
| Death | 3.75 (3.53, 3.97) | < .001 | -1.28 (-1.36, -1.19) | < .001 | 0.89 (-2.94, 4.72) | .776 | -1.02 (-1.84, -0.19) | .158 |
| Informal Speech | 3.19 (2.84, 3.53) | < .001 | 0.65 (0.51, 0.79) | < .001 | 2.93 (0.81, 5.04) | .059 | -0.28 (-0.97, 0.46) | .754 |
| Swear Words | 1.90 (1.34, 2.46) | < .001 | -0.68 (-0.92, -0.43) | < .001 | 2.60 (-1.83, 7.03) | .427 | -1.64 (-3.24, -0.21) | .158 |
| Netspeak | 4.34 (3.66, 5.03) | < .001 | 1.69 (1.37, 2.05) | < .001 | 2.91 (0.05, 5.77) | .183 | -0.60 (-1.62, 0.51) | .576 |
| Assent | 2.71 (2.06, 3.37) | < .001 | 0.03 (-0.20, 0.28) | .859 | 2.34 (-4.52, 9.19) | .636 | 1.36 (-0.30, 4.27) | .482 |
| Nonfluencies | 3.22 (2.46, 3.98) | < .001 | 1.05 (0.75, 1.39) | < .001 | 4.17 (-1.06, 9.39) | .274 | -0.23 (-1.54, 1.32) | .871 |
| Filler | 1.12 (-1.20, 3.44) | .365 | 0.29 (-0.43, 1.23) | .518 | - | - | - | - |
| All Punctuation | -1.97 (-2.35, -1.60) | < .001 | 0.33 (-0.06, 0.68) | .093 | 0.79 (-2.62, 4.19) | .784 | 1.51 (-0.31, 3.55) | .351 |
| Periods | 2.85 (2.72, 2.98) | < .001 | -0.14 (-0.33, 0.04) | .163 | 2.15 (0.85, 3.45) | .013 | 0.60 (-0.26, 1.41) | .457 |
| Commas | 0.34 (0.05, 0.63) | .023 | 1.11 (1.04, 1.18) | < .001 | 0.82 (-0.70, 2.34) | .521 | 0.70 (0.19, 1.22) | .158 |
| Colons | 1.44 (0.42, 2.46) | .007 | 2.19 (1.81, 2.62) | < .001 | 1.71 (-0.87, 4.30) | .411 | 1.36 (-0.30, 4.27) | .482 |
| Semicolons | 1.34 (0.36, 2.33) | .009 | 1.44 (1.04, 1.90) | < .001 | - | - | - | - |
| Question Marks | 2.84 (1.88, 3.79) | < .001 | 0.55 (0.24, 0.89) | .001 | 1.90 (-1.96, 5.76) | .489 | -0.16 (-1.72, 1.80) | .948 |
| Exclamation Marks | 4.27 (2.90, 5.64) | < .001 | -0.62 (-1.00, -0.19) | .003 | -4.03 (-10.1, 2.02) | .343 | -1.81 (-3.80, -0.16) | .197 |
| Dashes | 1.76 (1.04, 2.48) | < .001 | 1.64 (1.48, 1.82) | < .001 | 1.91 (-4.54, 8.36) | .717 | 0.15 (-0.61, 0.99) | .871 |
| Quotation Marks | 5.37 (4.33, 6.41) | < .001 | 1.14 (0.96, 1.34) | < .001 | 5.76 (-1.98, 13.5) | .343 | 0.0.25 (-0.64, 1.28) | .871 |
| Apostrophes | 3.52 (3.30, 3.74) | < .001 | -0.31 (-0.38, -0.23) | < .001 | 3.55 (1.27, 5.83) | .023 | 0.18 (-0.37, 0.75) | .843 |
| Parentheses (Pairs) | 2.32 (1.30, 3.33) | < .001 | 2.30 (2.08, 2.55) | < .001 | 3.39 (-0.43, 7.22) | .227 | 0.74 (-0.10, 1.75) | .364 |
| Other Punctuation | 0.09 (-0.83, 1.00) | .859 | 1.67 (1.54, 1.81) | < .001 | -0.75 (-8.24, 6.73) | .884 | 0.89 (0.15, 1.76) | .158 |
| TF-IDF^d^ |  |  |  |  | - | - | - | - |
| become obese | -0.03 (-0.07, 0) | .056 | 0.50 (0.08, 0.98) | .034 | - | - | - | - |
| lose weight | 0.00 (-0.05, 0.05) | .949 | 1.18 (0.81, 1.60) | < .001 | - | - | - | - |
| morbid obesity | 0.00 (-0.01, 0.02) | .573 | 0.11 (-0.17, 0.42) | .495 | - | - | - | - |
| morbidly obese | 0.08 (0.03, 0.14) | .003 | 0.28 (0, 0.58) | .068 | - | - | - | - |
| obese people | 0.05 (0.02, 0.08) | .003 | 0.65 (0.45, 0.86) | < .001 | - | - | - | - |
| obese person | -0.02 (-0.10, 0.05) | .538 | 1.48 (0.91, 2.17) | < .001 | - | - | - | - |
| obese woman | 0.03 (-0.12, 0.18) | .731 | 1.27 (-0.24, 4.14) | .235 | - | - | - | - |
| obese women | -0.02 (-0.16, 0.11) | .780 | 2.37 (1.41, 3.76) | < .001 | - | - | - | - |
| obesity epidemic | 0.01 (0, 0.02) | .032 | -1.69 (-1.82, -1.56) | < .001 | - | - | - | - |
| overweight obese | 0.01 (-0.02, 0.04) | .365 | 1.65 (1.40, 1.93) | < .001 | - | - | - | - |
| ^a^ CI: Confidence Interval  ^b^ For all semicontinuous models, the predictor is a categorical variable that denotes the word label of either fact (reference) or misinformation. The outcome is the value associated with the psycholinguistic feature of interest, truncated at zero. *P-*values are adjusted based on the Benjamini Hochberg Procedure.  ^c^ For all logistic regression models, the predictor is a categorical variable that denotes the word label of either fact (reference) or misinformation. The outcome is a binary variable of either 0 (if the value for the psycholinguistic feature is above zero) or 1 (if the value of the psycholinguistic feature is zero). *P-*values are adjusted based on the Benjamini Hochberg Procedure.  ^d^ TF-IDF: Term Frequency – Inverse Document Frequency  ^e^ VADER: Valence Aware Dictionary and SEntiment Reasoner  ^f^ LIWC: Linguistic Inquiry and Word Count Program | | | | | | | | |
