# Supplementary material for: Characterizing the Prevalence of Obesity Misinformation, Factual Content, Stigma, and Positivity on the Social Media Platform Reddit Between 2011 and 2019: Infodemiology Study
Source: J Med Internet Res. 2022 Dec 30;24(12):e36729. doi: 10.2196/36729 (PMC9840103; doi:10.2196/36729)
Supplement: Multimedia Appendix 11 [file jmir_v24i12e36729_app11.docx]

**Multimedia Appendix 11. Full Hurdle Models: Positivity vs. Stigma**

|  | Full Data | | | | Labeled Data | | | |
| --- | --- | --- | --- | --- | --- | --- | --- | --- |
|  | Semicontinuous Model | | Logistic Model | | Semicontinuous Model | | Logistic Model | |
| Outcome | β (95% CI^a^) | *P-*value^b^ | Log-Odds (95% CI) | *P-*value^c^ | β (95% CI) | *P-*value^b^ | Log-Odds (95% CI) | *P-*value^c^ |
| VADER^e^ |  |  |  |  |  |  |  |  |
| Negative Sentiment | 0.06 (0.05, 0.06) | < .001 | -0.85 (-0.91, -0.80) | < .001 | 0.06 (0.02, 0.09) | .021 | -0.37 (-0.89, 0.16) | .324 |
| Positive Sentiment | -0.02 (-0.03, -0.02) | < .001 | 0.15 (0.10, 0.20) | < .001 | -0.03 (-0.06, -0.01) | .125 | 0.73 (0.23, 1.25) | .041 |
| Compound Sentiment | -0.05 (-0.06, -0.04) | < .001 | 0.82 (0.78, 0.87) | < .001 | -0.13 (-0.23, -0.03) | .142 | 0.84 (0.34, 1.33) | .019 |
| LIWC^f^ |  |  |  |  |  |  |  |  |
| Language Metrics |  |  |  |  |  |  |  |  |
| Words Greater than Six Letters | 2.80 (2.63, 2.97) | < .001 | -0.83 (-1.00, -0.67) | < .001 | 3.33 (1.31, 5.35) | .036 | -0.70 (-1.77, 0.36) | .340 |
| Function Words |  |  |  |  |  |  |  |  |
| All Pronouns | -0.94 (-1.08, 0.80) | < .001 | 2.52 (2.19, 2.89) | < .001 | -0.69 (-2.39, 1.01) | .625 | 0.77 (-0.29, 2.05) | .338 |
| Personal Pronouns | -2.49 (-2.61, -2.38) | < .001 | 2.42 (2.25, 2.60) | < .001 | -0.72 (-2.17, 0.74) | .586 | 0.90 (0.12, 1.78) | .110 |
| First Person Singular Pronouns | -5.30 (-5.44, -5.16) | < .001 | 2.57 (2.51, 2.64) | < .001 | -2.32 (-3.92, -0.73) | .075 | 1.19 (0.67, 1.73) | < .001 |
| First Person Plural Pronouns | 1.05 (0.74, 1.36) | < .001 | -1.03 (-1.13, -0.92) | < .001 | 3.47 (-1.96, 8.89) | .510 | -0.18 (-1.26, 0.80) | .888 |
| Second Person Pronouns | 0.88 (0.67, 1.10) | < .001 | -0.86 (-0.91, -0.81) | < .001 | -0.62 (-3.59, 2.36) | .795 | -0.16 (-0.74, 0.39) | .770 |
| Third Person Singular Pronouns | 0.82 (0.54, 1.10) | < .001 | -1.01 (-1.09, -0.93) | < .001 | 0.96 (-1.53, 3.45) | .641 | -0.60 (-1.29, 0.04) | .205 |
| Third Person Plural Pronouns | 1.95 (1.74, 2.15) | < .001 | -2.12 (-2.19, -2.05) | < .001 | 1.86 (-0.55, 4.27) | .406 | -1.05 (-1.77, -0.39) | .041 |
| Impersonal Pronouns | 1.45 (1.35, 1.56) | < .001 | -0.73 (-0.78, -0.67) | < .001 | 1.33 (.06, 2.60) | .275 | 0.25 (-0.24, 0.75) | .509 |
| Articles | 0.64 (0.56, 0.71) | < .001 | -0.16 (-0.22, -0.11) | < .001 | 1.08 (-0.04, 2.19) | .295 | 0.08 (-0.42, 0.58) | .908 |
| Prepositions | -1.58 (-1.70, -1.46) | < .001 | 0.79 (0.67, 0.92) | < .001 | -1.36 (-2.67, -0.05) | .275 | 0.54 (-0.27, 1.44) | .373 |
| Auxiliary Verbs | -0.69 (-0.81, -0.57) | < .001 | 0.76 (0.62, 0.90) | < .001 | -0.65 (-2.07, 0.77) | .601 | 1.21 (0.08, 2.69) | .176 |
| Common Adverbs | -0.61 (-0.71, -0.50) | < .001 | 0.32 (0.27, 0.38) | < .001 | 0.60 (-0.52, 1.72) | .567 | 0.43 (-0.10, 0.99) | .252 |
| Conjunctions | 0.07 (-0.03, 0.16) | .216 | 0.68 (0.58, 0.79) | < .001 | 0.98 (-0.19, 2.14) | .364 | 0.90 (0.12, 1.78) | .110 |
| Negations | 0.32 (0.24, 0.40) | < .001 | -0.24 (-0.29, -0.19) | < .001 | 0.25 (-1.28, 1.78) | .836 | 0.43 (-0.05, 0.92) | .205 |
| Other Grammar |  |  |  |  |  |  |  |  |
| Regular Verbs | -1.92 (-2.06, -1.77) | .535 | 2.06 (1.41, 2.82) | < .001 | -1.26 (-2.90, 0.38) | .406 | 0.40 (-1.15, 2.36) | .810 |
| Adjectives | 0.04 (-0.08, 0.16) | < .001 | 0.26 (0.20, 0.33) | < .001 | -0.82 (-2.37, 0.72) | .567 | 0.81 (0.22, 1.44) | .058 |
| Comparatives | -0.42 (-0.52, -0.32) | < .001 | 0.35 (0.30, 0.39) | < .001 | -1.24 (-2.72, 0.24) | .364 | 0.70 (0.22, 1.19) | .041 |
| Interrogatives | 0.75 (0.67, 0.83) | < .001 | -0.63 (-0.68, -0.58) | < .001 | 0.50 (-0.56, 1.55) | .601 | -0.39 (-0.91, 0.12) | .287 |
| Numbers | -1.49 (-1.62, -1.36) | < .001 | 0.88 (0.83, 0.93) | < .001 | -2.15 (-3.96, -0.34) | .159 | 0.67 (0.15, 1.20) | .060 |
| Quantifiers | 0.22 (0.13, 0.30) | < .001 | -0.08 (-0.12, -0.03) | .001 | -0.62 (-1.61, 0.37) | .516 | 0.44 (-0.04, 0.92) | .205 |
| Affect Words | 1.72 (1.61, 1.83) | < .001 | -0.63 ( -0.70, -0.56) | < .001 | 1.04 (-0.39, 2.46) | .442 | -0.06 (-0.62, 0.51) | .945 |
| Positive Emotion | -0.28 (-0.38, -0.19) | < .001 | 0.17 (0.12, 0.21) | < .001 | -0.08 (-1.30, 1.14) | .932 | 0.65 (0.17, 1.14) | .548 |
| Negative Emotion | 2.00 (1.90, 2.10) | < .001 | -0.90 (-0.95, -0.85) | < .001 | 1.82 (0.35, 3.28) | .142 | -0.55 (-1.04, -0.07) | .098 |
| Anxiety | 0.36 (0.23, 0.49) | < .001 | -0.02 (-0.09, 0.06) | .670 | 1.30 (-1.73, 4.34) | .601 | -0.07 (-0.92, 0.73) | .959 |
| Anger | 1.95 (1.79, 2.11) | < .001 | -2.02 (-2.08, -1.96) | < .001 | 2.86 (1.29, 4.43) | .021 | -0.75 (-1.32, -0.20) | .058 |
| Sadness | -0.18 (-0.29, -0.08) | .001 | 1.04 (0.99, 1.10) | < .001 | -0.38 (-1.69, 0.93) | .739 | 0.57 (-0.09, 1.22) | .214 |
| Social Words | 5.69 (5.53, 5.85) | < .001 | -2.95 (-3.04, -2.86) | < .001 | 1.58 (-0.35, 3.50) | .376 | -1.33 (-1.93, -0.75) | < .001 |
| Family | 0.27 (0.10, 0.43) | .002 | -0.19 (-0.28, -0.11) | < .001 | 2.33 (-1.80, 6.45) | .528 | -0.77 (-2.05, 0.29) | .338 |
| Friends | 0.41 (0.27, 0.56) | < .001 | -0.50 (-0.59, -0.41) | < .001 | 1.98 (-0.23, 4.18) | .336 | 0.57 (-0.36, 1.49) | .382 |
| Female Referents | 1.04 (0.80, 1.28) | < .001 | -1.30 (-1.37, -1.23) | < .001 | 1.71 (-0.73, 4.15) | .450 | -0.71 (-1.38, -0.09) | .110 |
| Male References | 0.51 (0.29, 0.73) | < .001 | -0.87 (-0.95, -0.79) | < .001 | 2.00 (-1.25, 5.24) | .516 | -0.15 (-0.89, 0.55) | .849 |
| Cognitive Processes | 0.72 (0.56, 0.88) | < .001 | -0.18 (-0.28, -0.07) | .001 | 0.53 (-1.36, 2.43) | .748 | 0.24 (-0.50, 1.04) | .753 |
| Insight | -0.04 (-0.12, 0.05) | .438 | 0.14 (0.09, 0.18) | < .001 | 0.93 (-0.31, 2.17) | .415 | 0.65 (0.16, 1.14) | .058 |
| Cause | 0.20 (0.12, 0.29) | < .001 | -0.15 (-0.20, -0.11) | < .001 | 1.22 (-0.11, 2.54) | .334 | -0.44 (-0.96, 0.07) | .225 |
| Discrepancies | 0.12 (0.03, 0.21) | .013 | -0.14 (-0.19, -0.09) | < .001 | -0.31 (-1.80, 1.18) | .795 | 0.28 (-0.22, 0.77) | .443 |
| Tentativeness | 0.74 (0.65, 0.83) | < .001 | -0.35 (-0.40, -0.30) | < .001 | 1.16 (-0.46, 2.78) | .442 | -0.36 (-0.85, 0.12) | .287 |
| Certainty | 0.39 (0.31, 0.48) | < .001 | -0.32 (-0.37, -0.28) | < .001 | -0.68 (-1.75, 0.40) | .516 | 0.14 (-0.37, 0.64) | .786 |
| Differentiation | 0.19 (0.08, 0.29) | <.001 | 0.08 (0.03, 0.13) | .003 | 0.80 (-0.57, 2.17) | .528 | 0.16 (-0.33, 0.66) | .745 |
| Perceptual Processes | -0.08 (-0.16, 0) | .060 | 0.33 (0.29, 0.38) | < .001 | -0.23 (-1.52, 1.05) | .826 | 0.11 (-0.37, 0.59) | .822 |
| Seeing | 0.58 (0.47, 0.69) | < .001 | -0.67 (-0.73, -0.61) | < .001 | 0.79 (-1.01, 2.59) | .601 | -0.87 (-1.58, -0.23) | .059 |
| Hearing | 0.61 (0.48, 0.73) | < .001 | -0.78 (-0.85, -0.70) | < .001 | 0.56 (-1.78, 2.87) | .784 | -0.07 (-0.92, 0.73) | .959 |
| Feeling | -0.79 (-0.88, -0.69) | < .001 | 1.33 (1.28, 1.39) | < .001 | -0.73 (-2.33, 0.88) | .601 | 1.35 (0.75, 1.97) | < .001 |
| Biological Processes |  |  |  |  |  |  |  |  |
| Body | 0.72 (0.62, 0.82) | < .001 | -0.81 (-0.86, -0.76) | < .001 | 0.09 (-1.56, 1.74) | .932 | -0.55 (-1.13, 0.01) | .176 |
| Health | -0.30 (-0.39, -0.21) | < .001 | -0.66 (-3.73, 1.68) | .612 | - | - | - | - |
| Sexuality | 1.26 (1.03, 1.48) | < .001 | -1.75 (-1.84, -1.66) | < .001 | 1.40 (-0.89, 3.69) | .516 | -0.59 (-1.49, 0.22) | .327 |
| Ingestion | -0.68 (-0.77, -0.59) | < .001 | 0.03 (-3.20, 3.26) | .995 | - | - | - | - |
| Core Drives and Needs | -0.54 (-0.65, -0.43) | < .001 | 0.50 (0.43, 0.56) | < .001 | 0.32 (-1.10, 1.75) | .795 | 0.28 (-0.26, 0.84) | .509 |
| Affiliation | 0.38 (0.25, 0.51) | < .001 | -0.40 (-0.46, -0.35) | < .001 | 0.74 (-1.22, 2.69) | .646 | 0 (-0.60, 0.57) | .999 |
| Achievement | -0.97 (-1.06, -0.87) | < .001 | 1.11 (1.06, 1.16) | < .001 | 0.06 (-1.35, 1.46) | .950 | 0.61 (0.11, 1.11) | .072 |
| Power | 0.40 (0.32, 0.49) | < .001 | -0.12 (-0.16, -0.07) | < .001 | 0.71 (-0.90, 2.32) | .601 | 0.20 (-0.29, 0.69) | .623 |
| Reward Focus | -0.40 (-0.49, -0.31) | < .001 | 0.47 (0.42, 0.51) | < .001 | -0.13 (-1.74, 1.48) | .931 | 0.13 (-0.41, 0.67) | .809 |
| Risk/Prevention Focus | -0.17 (-0.26, -0.07) | .001 | 0.59 (0.53, 0.64) | < .001 | 0.88 (-0.66, 2.42) | .528 | 0.54 (-0.16, 1.24) | .267 |
| Time Orientation |  |  |  |  |  |  |  |  |
| Past Focus | -2.36 (-2.50, -2.23) | < .001 | 1.42 (1.37, 1.47) | < .001 | -0.78 (-2.32, 0.75) | .573 | 0.50 (0.01, 0.99) | .141 |
| Present Focus | 0.65 (0.51, 0.79) | < .001 | -0.49 (-0.63, -0.36) | < .001 | -0.13 (-1.79, 1.51) | .931 | 0.56 (-0.36, 1.60) | .432 |
| Future Focus | -0.04 (-0.14, 0.06) | .491 | 0.03 (-0.02, 0.08) | .314 | 0.81 (-1.38, 2.99) | .647 | -0.21 (-0.85, 0.41) | .745 |
| Relativity | -4.61 (-4.77, -4.45) | < .001 | 1.45 (1.36, 1.54) | < .001 | -2.29 (-4.19, -0.39) | .156 | 1.03 (0.35, 1.79) | .041 |
| Motion | -0.42 (-0.51, -0.34) | < .001 | 0.46 (0.41, 0.51) | < .001 | 0.36 (-0.79, 1.52) | .708 | 0.16 (-0.38, 0.70) | .762 |
| Space | -0.90 (-1.00, -0.79) | < .001 | 0.59 (0.53, 0.64) | < .001 | -0.68 (-2.06, 0.71) | .586 | 0.65 (0.13, 1.19) | .072 |
| Time | -3.02 (-3.14, -2.90) | < .001 | 1.20 (1.15, 1.25) | < .001 | -2.53 (-4.10, -0.97) | .036 | 0.34 (-0.14, 0.83) | .324 |
| Personal Concerns |  |  |  |  |  |  |  |  |
| Work | 0.08 (-0.02, 0.19) | .145 | 0.27 (0.22, 0.32) | < .001 | 1.51 (-0.00, 3.02) | .282 | 0.62 (0.09, 1.15) | .091 |
| Leisure | -0.15 (-0.26, -0.04) | .012 | 0.13 (0.07, 0.19) | < .001 | -0.01 (-1.32, 1.31) | .993 | 0.10 (-0.56, 0.74) | .908 |
| Home | 0.29 (0.13, 0.45) | .001 | -0.36 (-0.47, -0.26) | < .001 | -0.24 (-1.31, 0.84) | .795 | 0.02 (-1.09, 1.04) | .999 |
| Money | 0.79 (0.61, 0.96) | < .001 | -0.88 (-0.96, -0.80) | < .001 | 0.27 (-2.93, 3.47) | .931 | -1.34 (-2.81, -0.23) | .117 |
| Religion | 0.74 (0.45, 1.02) | < .001 | -1.02 (-1.16, -0.89) | < .001 | 2.86 (-36.4, 42.1) | .708 | -0.17 (-3.25, 2.19) | .959 |
| Death | 0.62 (0.37, 0.86) | < .001 | -1.06 (-1.19, -0.93) | < .001 | -0.30 (-5.53, 4.93) | .932 | -1.32 (-3.19, 0.01) | .214 |
| Informal Speech | 1.48 (1.35, 1.62) | < .001 | -1.24 (-1.29, -1.19) | < .001 | 1.39 (-0.19, 2.98) | .336 | -0.72 (-1.30, -0.18) | .059 |
| Swear Words | 1.83 (1.62, 2.04) | < .001 | -2.12 (-2.20, -2.05) | < .001 | 1.81 (-0.25, 3.88) | .336 | -1.06 (-1.81, -0.38) | .041 |
| Netspeak | 0.07 (-0.08, 0.23) | .378 | 0.07 (-0.02, 0.16) | .145 | 1.36 (-1.32, 4.04) | .567 | -0.46 (-1.62, 0.55) | .609 |
| Assent | 0.31 (0.14, 0.49) | .001 | -0.23 (-0.33, -0.14) | < .001 | -3.33 (-6.81, 0.16) | .295 | 0.13 (-0.99, 1.18) | .938 |
| Nonfluencies | -0.13 (-0.31, 0.05) | .172 | 0.13 (0.02, 0.25) | .028 | 2.16 (-2.30, 6.61) | .567 | -0.17 (-1.74, 1.18) | .398 |
| Filler | 0.30 (-0.16, 0.76) | .230 | -0.08 (-0.34, 0.19) | .607 | - | - | - | - |
| All Punctuation | -1.06 (-1.25, -0.86) | < .001 | 1.34 (0.93, 1.77) | < .001 | 0.79 (-2.08, 3.67) | .748 | - | - |
| Periods | 0.15 (0.08, 0.22) | < .001 | 0.45 (0.37, 0.52) | < .001 | 1.04 (-0.28, 2.35) | .406 | 0 (-0.72, 0.76) | .999 |
| Commas | 0.76 (0.64, 0.87) | < .001 | 0.25 (0.20, 0.30) | < .001 | 2.07 (-0.00, 4 .415) | .282 | 0.16 (-0.33, 0.65) | .745 |
| Colons | -0.09 (-0.32, 0.15) | .504 | 0.12 (0, 0.25) | .058 | -1.33 (-4.74, 2.08) | .625 | -0.26 (-1.34, 0.70) | .792 |
| Semicolons | 0.04 (-0.21, 0.29) | .749 | 0.28 (0.11, 0.44) | .001 | -2.73 (-20.8, 15.3) | .570 | -0.17 (-3.25, 2.19) | .959 |
| Question Marks | 0.97 (0.64, 1.30) | < .001 | -1.55 (-1.70, -1.40) | < .001 | 1.70 (-2.62, 6.01) | .606 | -0.04 (-1.39, 1.19) | .999 |
| Exclamation Marks | -0.39 (-0.76, -0.01) | .052 | 0.55 (0.44, 0.67) | < .001 | -0.84 (-6.58, 4.80) | .837 | 0.80 (-0.16, 1.79) | .233 |
| Dashes | 0.29 (0.13, 0.45) | .001 | 0.06 (0, 0.13) | .068 | 1.63 (-1.16, 4.41) | .528 | 0.02 (-0.77, 0.76) | .999 |
| Quotation Marks | 0.02 (-0.24, 0.28) | .873 | -0.26 (-0.33, -0.19) | < .001 | 0.69 (-3.38, 4.77) | .828 | 0.67 (-0.09, 1.420 | .208 |
| Apostrophes | -0.31 (-0.41, -0.20) | < .001 | 0.27 (0.22, 0.31) | < .001 | 0.58 (-1.07, 2.23) | .672 | 0.44 (-0.04, 0.92) | .205 |
| TF-IDF^d^ |  |  |  |  | - | - | - | - |
| become obese | 0.01 (0.00, 0.03) | .172 | 0.11 (-0.08, 0.31) | .265 | - | - | - | - |
| com url | 0.06 (-0.31, 0.44) | .749 | 2.37 (0.73, 5.27) | .027 | - | - | - | - |
| lose weight | -0.07 (-0.09, 0.06) | < .001 | 1.79 (1.67, 1.91) | < .001 | - | - | - | - |
| morbid obesity | 0.01 (0, 0.03) | .065 | 0.25 (0.02, 0.49) | .037 | - | - | - | - |
| morbidly obese | -0.02 (-0.03, -0.01) | .001 | -0.98 (-1.05, -0.91) | < .001 | - | - | - | - |
| obese people | 0.06 (0.04, 0.08) | < .001 | -1.80 (-1.92, -1.67) | < .001 | - | - | - | - |
| obese person | 0.00 (-0.02, 0.02) | .836 | -1.04 (-1.22, -0.88) | < .001 | - | - | - | - |
| obese woman | -0.01 (-0.04, 0.02 | .581 | -1.81 (-2.17, -1.48) | < .001 | - | - | - | - |
| obese women | 0.04 (0, 0.08) | .061 | -2.79 (-3.27, -2.36) | < .001 | - | - | - | - |
| obesity epidemic | 0.00 (-0.03, 0.03) | .839 | -0.75 (-1.16, -0.37) | < .001 | - | - | - | - |
| ^a^ CI: Confidence Interval  ^b^ For all semicontinuous models, the predictor is a categorical variable that denotes the word label of either positivity (reference) or stigma. The outcome is the value associated with the psycholinguistic feature of interest, truncated at zero. *P-*values are adjusted based on the Benjamini Hochberg Procedure.  ^c^ For all logistic regression models, the predictor is a categorical variable that denotes the word label of either positivity (reference) or stigma. The outcome is a binary variable of either 0 (if the value for the psycholinguistic feature is above zero) or 1 (if the value of the psycholinguistic feature is zero). *P-*values are adjusted based on the Benjamini Hochberg Procedure.  ^d^ TF-IDF: Term Frequency – Inverse Document Frequency  ^e^ VADER: Valence Aware Dictionary and SEntiment Reasoner  ^f^ LIWC: Linguistic Inquiry and Word Count Program | | | | | | | | |
